# Regioselective hydroesterification of alkenes and alkenylphenols utilizing CO_2_ and hydrosilane†

**DOI:** 10.1039/d3sc01114c

**Published:** 2023-04-18

**Authors:** Meng-Meng Wang, Sheng-Mei Lu, Can Li

**Affiliations:** a State Key Laboratory of Catalysis, Dalian Institute of Chemical Physics, Chinese Academy of Sciences, Dalian National Laboratory for Clean Energy Dalian 116023 China canli@dicp.ac.cn; b University of Chinese Academy of Sciences Beijing 100049 China

## Abstract

As an important and attractive C1 building block, the diversified exploitation of CO_2_ in chemical transformations possesses significant research and application value. Herein, an effective palladium-catalyzed intermolecular hydroesterification of a wide range of alkenes with CO_2_ and PMHS is described, successfully generating diverse esters with up to 98% yield and up to 100% linear-selectivity. In addition, the palladium-catalyzed intramolecular hydroesterification of alkenylphenols with CO_2_ and PMHS is also developed to construct a variety of 3-substituted-benzofuran-2(3*H*)-ones with up to 89% yield under mild conditions. In both systems, CO_2_ functions as an ideal CO source with the assistance of PMHS, thus smoothly participating in a series of alkoxycarbonylation processes.

## Abstract 2

Regioselective hydroesterification of alkenes and alkenylphenols was achieved utilizing CO_2_ as a CO source with the assistance of PMHS.

## Graphical abstract

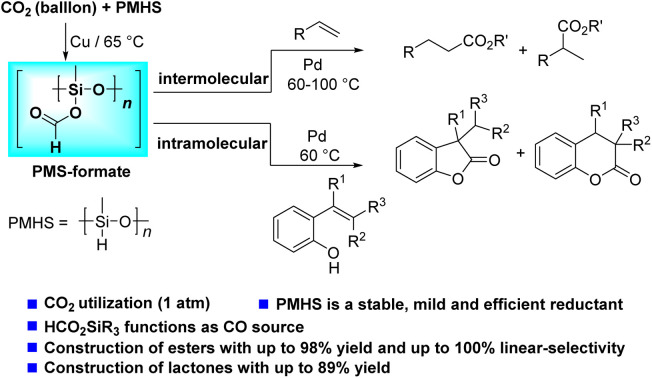

## Introduction

**Scheme 1:**
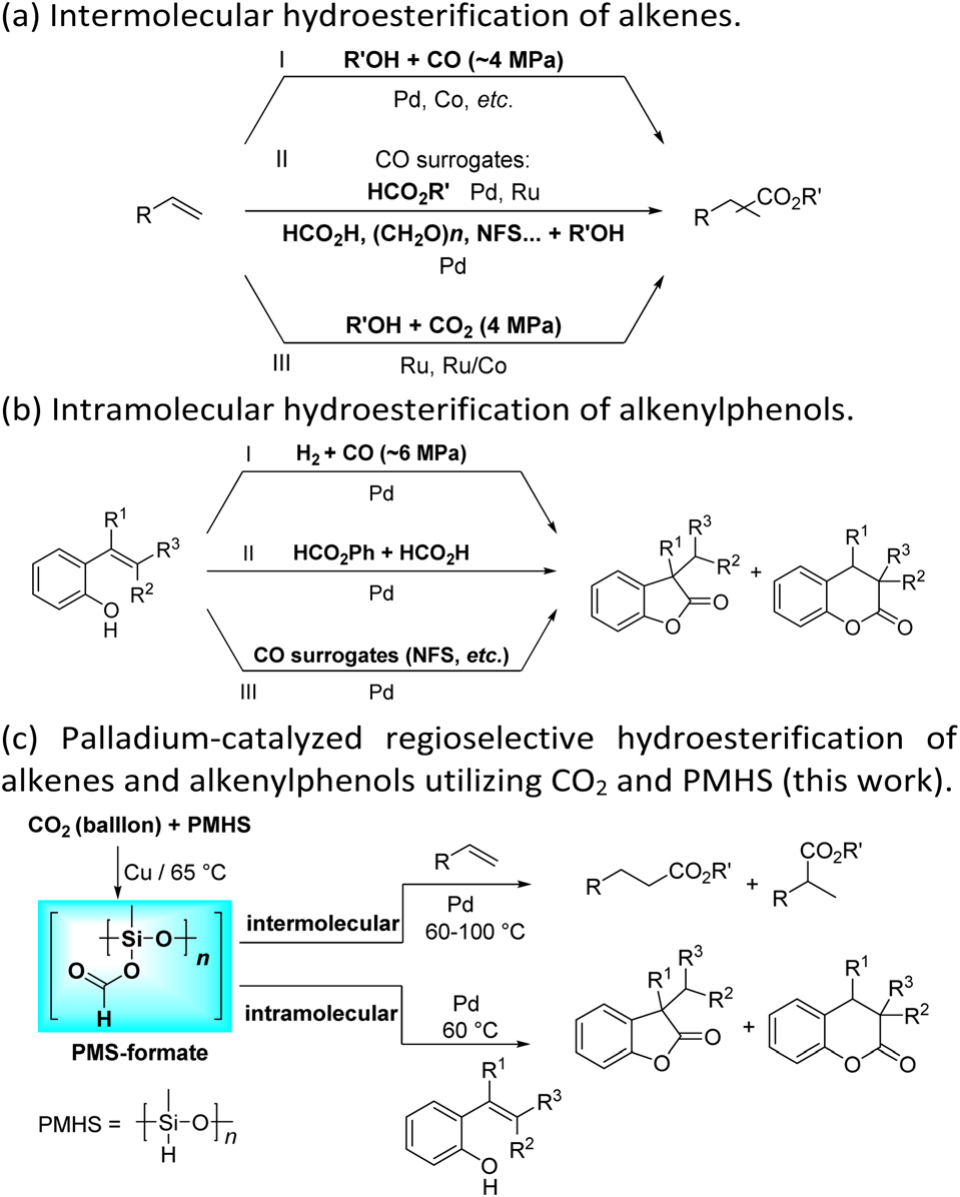
Transition-metal-catalyzed hydroesterification of alkenes and alkenylphenols.

Transition-metal-catalyzed hydroesterification reactions of alkenes represent powerful means for the production of esters and lactones, which are important classes of value-added bulk and fine chemicals and widely exist in various biologically active substances.^1–4^ Apparently, CO is an adept and abundant C1 synthon; thus until now the vast majority of the intermolecular hydroesterification of alkenes has prominently employed CO in the presence of alcohol (Scheme 1a(I)).^5–10^ However, gaseous CO with flammable and toxic properties sometimes limits its research use and exploration in laboratories. To avoid the direct use of external gaseous CO, continuous and substantial efforts have been made for the development of less toxic and easy-to-handle CO surrogates to facilitate “CO-free” carbonylation processes in the past few years.^11–25^ Using formates, including alkyl formates or phenyl formate, as the sources of CO and nucleophilic alcohol, the ruthenium- or palladium-catalyzed intermolecular hydroesterification of alkenes was developed (Scheme 1a(II)).^17–20^ Besides, Beller's group successfully converted a series of alkenes into esters by employing HCO_2_H as a CO source, where palladium catalysts containing elaborate ligands with a built-in base facilitate the selective decomposition of HCO_2_H to CO during the reaction (Scheme 1a(II)).^21^ Additionally, the application of paraformaldehyde ((CH_2_O)_*n*_) and *N*-formylsaccharin (NFS) as CO surrogates was also exploited to participate in intermolecular hydroesterification processes (Scheme 1a(II)).^22,23^

These years have witnessed the prevalence of CO_2_ in organic synthesis as an ideal and promising C1 synthon owing to its nontoxicity, abundance and sustainability.^26–28^ Undoubtedly, the CO_2_ molecule is a perfect candidate for the direct carboxylation of various organic compounds which can insert CO_2_ into the C–X bond as a whole *via* the carboxylation process.^29–32^ Additionally, with the assistance of suitable reductants, CO_2_ can be transformed into CO and thus smoothly participates in a variety of subsequent carbonylation reactions, which greatly expands its application range.^33–47^ In contrast with extensive research on the CO-based intermolecular hydroesterification of alkenes,^5–10^ the intermolecular hydroesterification of alkenes with CO_2_ has been scarcely studied. In 2014, Beller's group developed ruthenium-catalyzed hydroesterification reactions of alkenes with the utilization of CO_2_ and alcohols at 160 °C, during which the *in situ* formation of CO from CO_2_ and alcohols allows for an effective synthesis of esters (Scheme 1a(III)).^48^ After this, He's group realized the intermolecular hydroesterification of alkenes with CO_2_ and alcohols employing a bi-metal ruthenium/cobalt catalytic system, which effectively reduced the amount of precious metal Ru and ionic liquid in the reaction (Scheme 1a(III)).^49^

For the intramolecular form, the combination of CO and H_2_ can effectively achieve the intramolecular hydroesterification of alkenylphenols (Scheme 1b(I)).^3,4,50^ Besides, Shi's group accomplished the reactions with the assistance of phenyl formate and HCO_2_H (Scheme 1b(II)).^24,51^ Using NFS as a CO source, the intramolecular hydroesterification of alkenylphenols was also realized (Scheme 1b(III)).^25^ However, there is no example of intramolecular hydroesterification of alkenes using CO_2_ as a CO source.

Different reductants have been exploited to enable carbonylation processes using CO_2_ instead of CO as a C1 resource, among which H_2_ is the greenest and most renewable one as no concern about waste generation remains. However, these reactions inevitably face problems such as poor selectivity caused by harsh conditions (high temperature and/or high pressure).^35,38–40^ Therefore, the design and implementation of mild and efficient reduction systems become more crucial to further improve the utility of CO_2_ in carbonylation processes. PMHS (polymethylhydrosiloxane), the byproduct of the silicone industry, is a kind of cheap, stable and readily available reductant.^52^ Compared with the high energy demand for the transformation of CO_2_ with H_2_, the hydrosilylation of CO_2_ with hydrosilane is an exothermic reaction, which is thermodynamically favorable.^53^ Consequently, PMHS can convert CO_2_ into silyl formate in a quite mild manner.^54,55^ Furthermore, silyl formate could be easily decomposed into CO and silanol, thus realizing the convenient transformation from CO_2_ to CO. Our group has been committed to making use of the combination CO_2_ and PMHS to realize the carbonylation of organic compounds. More specifically, the silyloxycarbonylation of aryl halides, the hydrocarboxylation of alkynes and the intramolecular Heck carbonylation of alkenes have been successfully implemented, affording various valued carbonyl-containing compounds.^36,47^ As part of our constant interest and endeavor in CO_2_ utilization, here we report palladium-catalyzed hydroesterification of alkenes using *in situ* formed silyl formate from CO_2_ and PMHS as a CO source (Scheme 1c). Both the intermolecular hydroesterification of alkenes and the intramolecular hydroesterification of alkenylphenols can be conducted efficiently and regioselectively, obtaining a variety of esters and lactones.

## Results and discussion

Initially, the intermolecular hydroesterification of styrene 1a was performed in a conventional glass reaction tube, where silyl formate was freshly synthesized *in situ* from PMHS and a CO_2_ balloon *via* the Cu(OAc)_2_/dppbz-catalyzed hydrosilylation process (Table 1). A catalyst system combining palladium precursor and bidentate phosphine ligand dtbpx was chosen for the subsequent hydroesterification reaction on the basis of their commercial availability and outstanding performance in laboratory research and industrial production.^56^ Besides, PTSA is the most commonly used acidic co-catalyst in this hydroesterification reaction to foster the most likely “hydride-cycle” pathway. Thus, the combination of Pd(OAc)_2_, dtbpx and PTSA was tentatively adopted to study the reactivity and regioselectivity of the hydroesterification of styrene in different alcohol solvents. In the tested primary and secondary alcohols, the reactions have an obvious preference for linear products in all cases, which originates from the linear Pd complex intermediate formed by the migratory insertion of an alkene into the Pd–H bond. The yields of the corresponding esters decreased from primary to secondary alcohols (Table 1, entries 1–4). Only trace ester was formed when it came to tertiary alcohol ^*t*^BuOH, which could be attributed to its severe steric hindrance that hinders alcoholysis of the acyl palladium(ii) species, the rate-determining step of the hydroesterification catalytic cycle (Table 1, entry 5).^1^ Additionally, the viscosity of solvent also plays a vital role in the CO gas-involved reaction, and stickiness of ^*i*^PrOH and ^*t*^BuOH may be partially responsible for the decreased activity. MeOH and EtOH show comparably excellent performance in the reaction, but considering the regioselectivity, EtOH with better linear selectivity (Table 1, entries 1–2, *l*/*b* = 3.8/1.0 *vs.* 2.7/1.0) was chosen as the solvent in the next reaction investigations.

**Table 1:** Alcohol solvent optimization of the intermolecular hydroesterification of styrene^a^

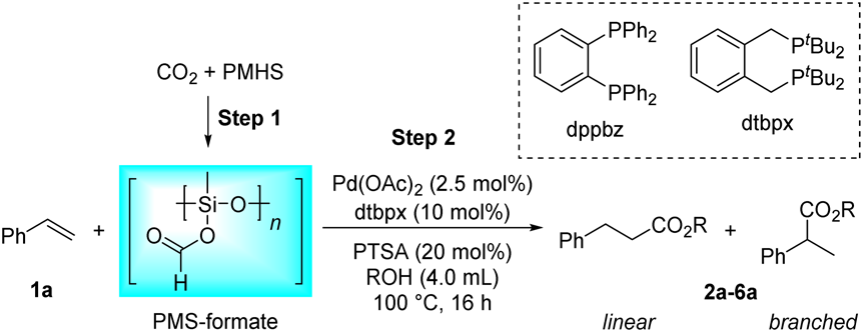				
Entry	Solvent	Product	Yield^b^ (%)	*l*/*b*^b^
1	MeOH	2a	92	2.7/1.0
2	EtOH	3a	94	3.8/1.0
3	^*n*^PrOH	4a	50	2.3/1.0
4	^*i*^PrOH	5a	20	4.5/1.0
5	^*t*^BuOH	6a	Trace	—

a Reaction conditions: step 1: Cu(OAc)_2_ (1.0 mol%), dppbz (1.5 mol%), PMHS (0.165 g, Si–H, 2.5 mmol), CO_2_ (balloon), dry 1,4-dioxane (0.5 mL), 65 °C, 30 min. Step 2: 1a (1.0 mmol), Pd(OAc)_2_ (2.5 mol%), dtbpx (10 mol%), PTSA (20 mol%), dry solvent (ROH, 4.0 mL), 100 °C, 16 h.

b Yields and selectivities were determined by ^1^H NMR using an internal standard.

Then, the influence of the acidic co-catalysts was evaluated (Table 2). Methanesulfonic acid, another widely used sulfonic acid in this type of reaction, can perform this reaction brilliantly with nearly parallel yield and selectivity compared to PTSA (Table 2, entry 2). Notably, using stronger Brønsted acids can greatly alter and even reverse the regioselectivity of the reaction. Specifically, the use of TFA resulted in a dramatic drop in activity and no preference for either linear or branched products appeared (Table 2, entry 3). Remarkably, in the reaction using racemic arylphosphonic acid BNPA or DPPA as an acidic co-catalyst, a totally reversed preference emerged, affording branched esters predominantly, albeit with low reactivity (Table 2, entries 4–5). As described above, the activity and regioselectivity of the hydroesterification of styrene have been proved to be essentially susceptible to the nature of the counteranion of the acid used. Weakly coordinating sulfonate anions were observed to favor the generation of linear esters. Based on these preliminary results, we believe that it is feasible to control the regioselectivity by a delicate and judicious adjustment of the acidic promoter, which will allow the flexibility of the research and production. The control experiment without any acid has affirmed that the acid is indispensable for the hydroesterification reaction (Table 2, entry 6).

**Table 2:** Acidic co-catalyst optimization of the intermolecular hydroesterification of styrene^a^

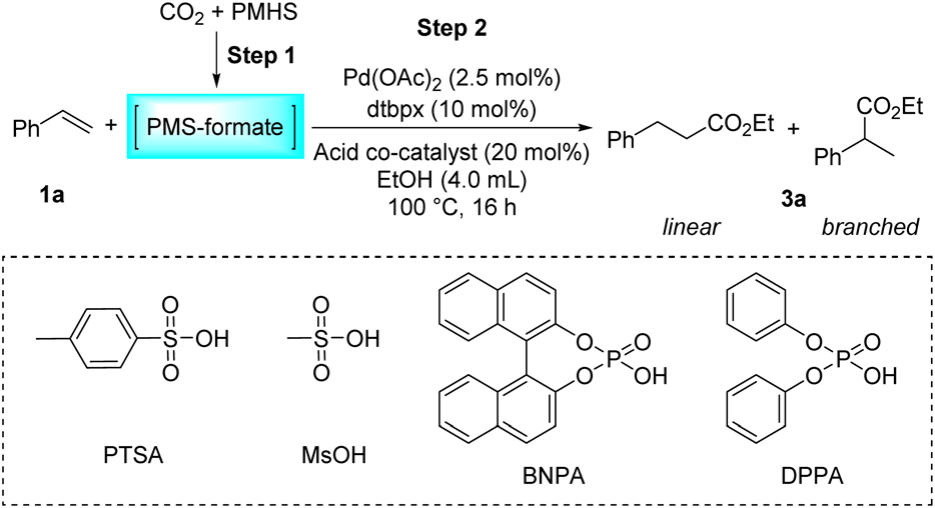			
Entry	HX	Yield^b^ (%)	*l*/*b*^b^
1	PTSA	94	3.8/1.0
2	MsOH	92	3.5/1.0
3	TFA	8	1.0/1.0
4	BNPA	38	1.0/1.9
5	DPPA	36	1.0/2.3
6	—	N.D.	—

a Reaction conditions: step 1: the same as that in Table 1. Step 2: 1a (1.0 mmol), Pd(OAc)_2_ (2.5 mol%), dtbpx (10 mol%), acid co-catalyst (20 mol%), dry EtOH (4.0 mL), 100 °C, 16 h.

b Yields and selectivities were determined by ^1^H NMR using an internal standard. TFA = trifluoroacetic acid. N.D. = not detected.

Furthermore, temperature and palladium precursor screenings were performed (Table 3). Reducing the temperature to 90 °C didn't affect the reactivity and slightly reduced the linear selectivity (*l*/*b* = 2.9/1.0) (Table 3, entry 2). When 80 °C was applied to the reaction, only moderate yield was obtained (71%) with obviously reduced selectivity (*l*/*b* = 2.1/1.0) (Table 3, entry 3). A lower reaction temperature of 60 °C led to a drastic decrease in activity (40%) and an inversion of selectivity (*l*/*b* = 1.0/1.2) (Table 3, entry 4). When the reaction time was extended to 48 h at 60 °C, the regioselectivity of the reaction remained virtually unchanged (*l*/*b* = 1.0/1.3), and the yield was greatly improved to 82% (Table 3, parentheses in entry 4). High temperatures contribute to the formation of a linear product presumably as the β-hydride elimination of the stabilized π-benzylic palladium species is facilitated under these conditions, allowing the reinsertion of the alkene into the Pd–H bond to generate the linear palladium alkyl species accordingly.^57^ When the temperature was increased to 120 °C, a yield of 83% was obtained with a selectivity of *l*/*b* = 4.8/1.0 (Table 3, entry 5). In this case, although increasing the temperature is beneficial to improve the selectivity, it is not conducive to the maintenance of the reactivity. This may be attributed to the reduced stability of the catalyst at too high temperature, as obvious palladium black was observed in the solution after the reaction at 120 °C. The evaluation of the palladium precursors has illustrated that Pd(acac)_2_ has considerably strong capability for this reaction, just like Pd(OAc)_2_ (Table 3, entries 1 and 6). However, the replacement of Pd(OAc)_2_ with Pd_2_(dba)_3_ resulted in a quite low yield (Table 3, entry 7). Besides, Pd(PPh_3_)_4_ and PdCl_2_ are totally invalid for this reaction (Table 3, entries 8–9).

**Table 3:** Temperature and palladium precursor optimization of the intermolecular hydroesterification of styrene^a^

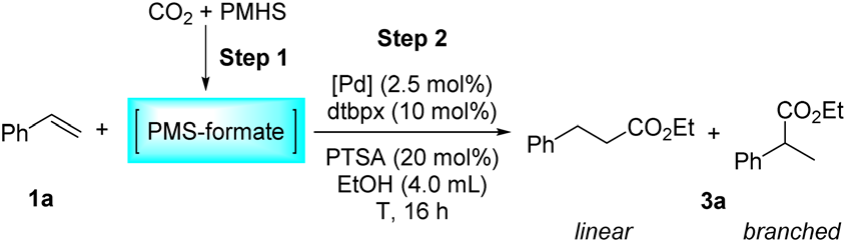				
Entry	Catalyst	*T*/°C	Yield^b^ (%)	*l*/*b*^b^
1	Pd(OAc)_2_	100	94	3.8/1.0
2	Pd(OAc)_2_	90	94	2.9/1.0
3	Pd(OAc)_2_	80	71	2.1/1.0
4	Pd(OAc)_2_	60	40 (82)^c^	1.0/1.2 (1.0/1.3)^c^
5	Pd(OAc)_2_	120	83	4.8/1.0
6	Pd(acac)_2_	100	94	3.6/1.0
7	Pd_2_(dba)_3_	100	21	3.7/1.0
8	Pd(PPh_3_)_4_	100	N.D.	—
9	PdCl_2_	100	N.D.	—

a Step 1: the same as that in Table 1. Step 2: 1a (1.0 mmol), [Pd] (2.5 mol%), dtbpx (10 mol%), PTSA (20 mol%), dry EtOH (4.0 mL), *T*, 16 h.

b Yields and selectivities were determined by ^1^H NMR using an internal standard.

c 60 °C, 48 h, isolated yield.

Compared with catalytic systems making use of different CO surrogates (Table S1†), this catalytic system employing atmospheric pressure CO_2_ as a CO source has outstanding advantages, such as ready availability, safety and high reactivity. Then, we continued to examine the reactivity profile of different styrene derivatives in the ethoxycarbonylation reactions (Table 4). The reactions of *para*-, *meta*- and *ortho*-methyl substituted styrene derivatives 1b–1d all proceeded excellently with high yields and linear selectivities (Table 4, entries 2–4). Among them, the *ortho*-methylstyrene 1d has the most prominent linear selectivity (*l*/*b* = 10.1/1.0), which is attributed to the steric hindrance of its *ortho*-methyl group that is more conducive to the generation of the linear product (Table 4, entry 4). However, 4-methoxystyrene 1e only gave a low yield of 40% as a noticeable amount of side product ether was formed from the competitive insertion of ethanol into alkene (Table 4, entry 5). When styrene derivative 1f bearing a *para*-chloro group on the phenyl ring was hydroesterified under the same conditions, only 19% yield (*l*/*b* = 2.6/1.0) was obtained due to the severe competitive hydrogenation of the substrate (Table 4, parentheses in entry 6). Thus, milder conditions (60 °C, 48 h) were adopted for the *para*-, *meta*- and *ortho*-chloro substituted styrene derivatives 1f–1h. Delightfully, lower temperature successfully suppressed the occurrence of side reactions and the corresponding esters 3f–3h were isolated in satisfactory yields (79–84%) (Table 4, entries 6–8). As expected, pronounced branched selectivities were achieved for *para*- and *meta*-chloro substituted substrates 1f and 1g as the formation of branched esters is favored at low reaction temperature (Table 4, entries 6–7). In contrast, the preference for the linear product is still maintained (*l*/*b* = 4.9/1.0) for *ortho*-chlorostyrene 1h even at 60 °C, indicating that when temperature and the steric hindrance factor coexist with opposite effects on selectivity, the effect of steric hindrance is dominant while the effect of temperature is almost negligible (Table 4, entry 8). For the styrene derivative 1i with a 4-phenyl substituent, a good yield of 80% was achieved with a selectivity of *l*/*b* = 2.3/1.0 (Table 4, entry 9). Moreover, the catalytic system is also feasible for 2-vinylnaphthalene 1j, affording the corresponding ester with moderate yield (67%) and good linear selectivity (*l*/*b* = 2.8/1.0) (Table 4, entry 10).

**Table 4:** The intermolecular hydroesterification of substituted vinyl arenes^a^

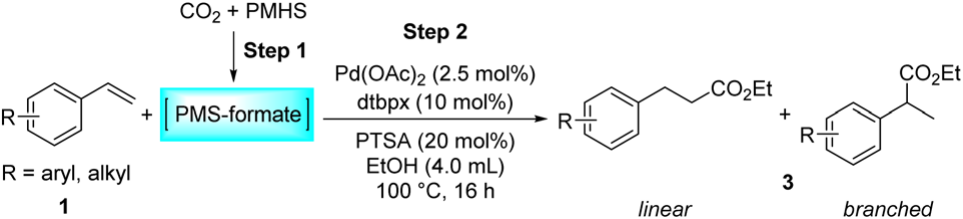			
Entry	Substrate	Yield^b^ (%)	*l*/*b*^c^
1	R = H, 1a	3a, 94	3.8/1.0
2	R = 4-Me, 1b	3b, 91	4.3/1.0
3	R = 3-Me, 1c	3c, 88	3.8/1.0
4	R = 2-Me, 1d	3d, 93	10.1/1.0
5	R = 4-OMe, 1e	3e, 40	4.9/1.0
6	R = 4-Cl, 1f	3f, 79^d^ (19)^a^	1.0/2.1^d^ (2.6/1.0)^a^
7	R = 3-Cl, 1g	3g, 84^e^	1.0/2.1^e^
8	R = 2-Cl, 1h	3h, 81^e^	4.9/1.0^e^
9	R = 4-Ph, 1i	3i, 80^f^	2.3/1.0^f^
10	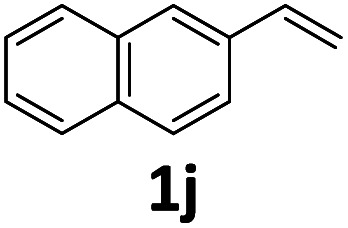	3j, 67^g^	2.8/1.0^g^

a Reaction conditions: step 1: the same as that in Table 1. Step 2: 1 (1.0 mmol), Pd(OAc)_2_ (2.5 mol%), dtbpx (10 mol%), PTSA (20 mol%), dry EtOH (4.0 mL), 100 °C, 16 h.

b Isolated yields.

c Selectivities were determined by ^1^H NMR.

d 60 °C, 48 h.

e 60 °C, 48 h, Pd(acac)_2_.

f 100 °C, 24 h.

g 100 °C, 24 h, Pd(acac)_2_.

Next, we turned our attention towards the reactivity of other types of alkenes in the intermolecular hydroesterification reactions (Table 5). To our delight, the terminal aliphatic alkene 1-octene (1k) was exclusively converted into the corresponding linear ethyl nonanoate (3k) in nearly quantitative yield (98%) (Table 5, entry 1). More remarkably, our catalytic system is also competent to selectively transform aliphatic internal alkene 2-octene (1l) into 3k with 95% yield and 100% linear-selectivity *via* isomerization and consecutive ethoxycarbonylation processes (Table 5, entry 2). Less expensive mixtures of terminal and internal alkenes are generally preferred as starting materials in bulk industrial carbonylation processes, and our carbonylation strategy has great potential to convert the mixtures into the single linear ethyl nonanoate in this regard. When methyl 2-pentenoate (1m) was subjected to the reaction using MeOH as solvent, it could be smoothly converted into sole linear ester-dimethyl adipate (2m), which is an important chemical raw material especially for the polymer industry (Table 5, entry 3). Notably, in this case even the double bond conjugated to the ester group could be isomerized to the terminal position of the carbon chain and readily go through the subsequent methoxycarbonylation transformation. The extraordinary selectivity of isomerizing alkoxycarbonylation of unsaturated fatty acid esters can be traced to two decisive points, which are responsible for the kinetically controlled formation of the linear α,ω-product: a preference for linear insertion products and the relatively slow methanolysis of the branched acyl palladium.^58,59^ Once again, when *N*-vinylphthalimide (1n) was investigated as a type of *N*-substituted alkene, linear ester 3n was exclusively produced in high yield (Table 5, entry 4). As expected, the reaction of α-methylstyrene (1o) with silyl formate solely generated terminal aliphatic ester 3o, albeit in moderate yield (Table 5, entry 5). Surprisingly, the transformations of both allyl benzene (1p) and β-methylstyrene (1q) provided terminal ethyl phenylbutyrate (3p) as the sole regioisomer, whereas 1q led to a lower yield than 1p due to the initial isomerization (Table 5, entries 6–7). Clearly, our strategy utilizing silyl formate *in situ* generated from CO_2_ and PMHS as CO sources enables an exclusive linear-selectivity for intermolecular hydroesterification reactions of a wide range of alkenes.

**Table 5:** The intermolecular hydroesterification of other alkene types^a^

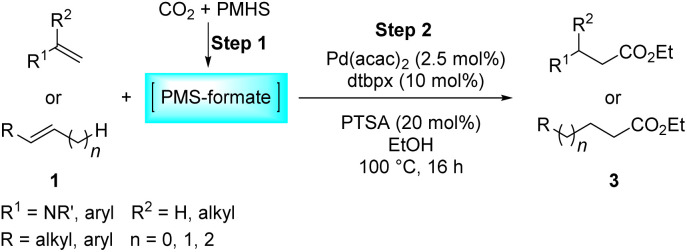			
Entry	Substrate	Product	Yield^b^ (%)
1	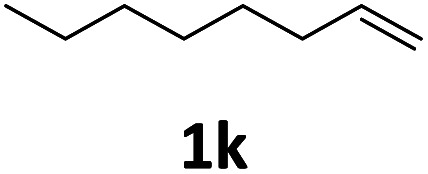	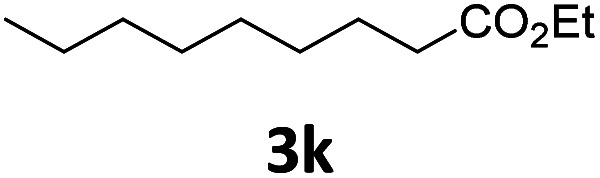	98
2	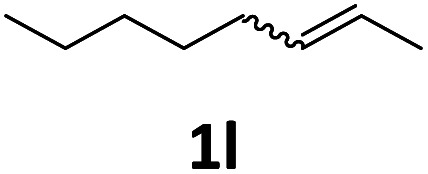	3k	95
3	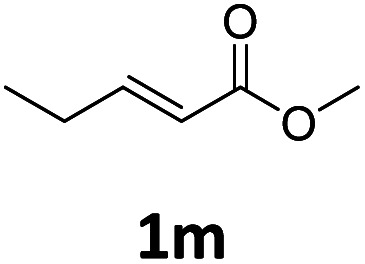	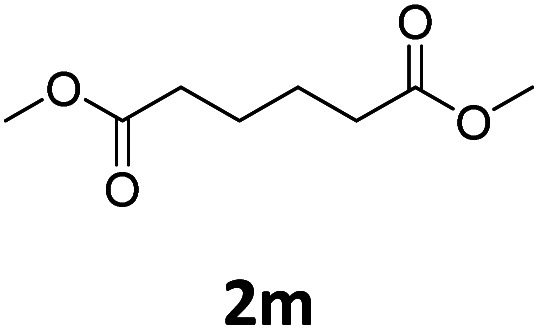	75^c^
4	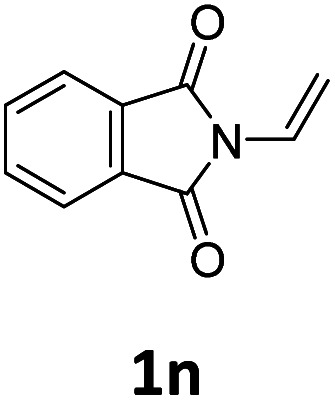	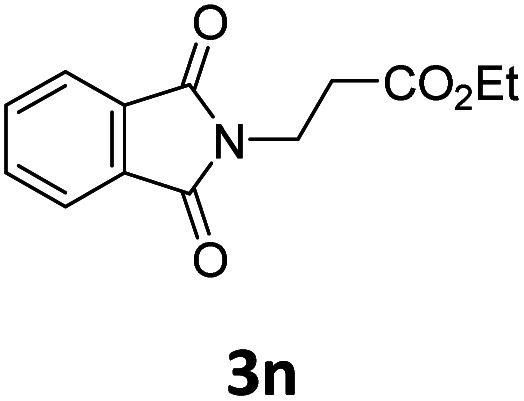	96
5	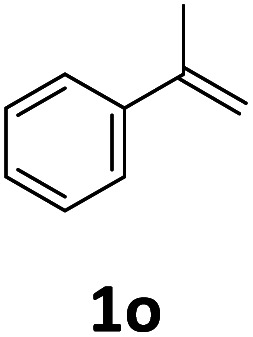	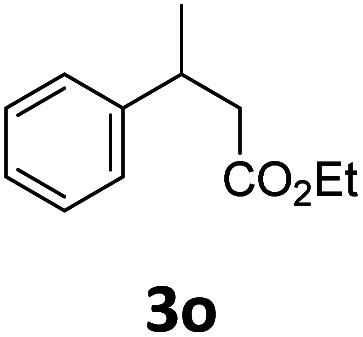	35^d^
6	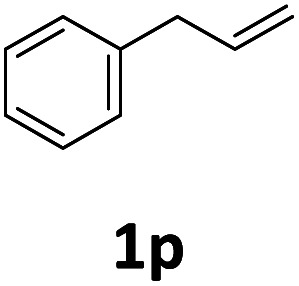	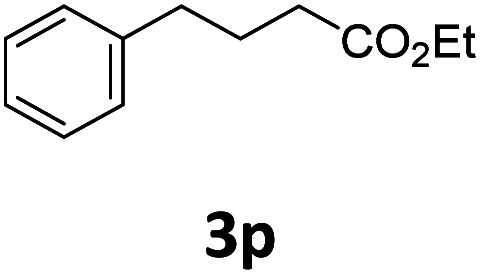	62
7	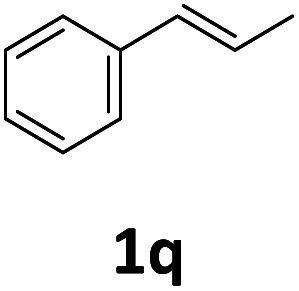	3p	42

a Reaction conditions: step 1: the same as that in Table 1. Step 2: 1 (1.0 mmol), Pd(acac)_2_ (2.5 mol%), dtbpx (10 mol%), PTSA (20 mol%), dry EtOH (4.0 mL), 100 °C, 24 h.

b Isolated yields.

c Dry MeOH (4.0 mL).

d 100 °C, 16 h.

Benzofuran-2(3*H*)-ones constitute an important part of natural structural moieties with distinct biological activities and are widely used in polymer chemistry, which can be synthesized through the intramolecular hydroesterification of alkenylphenols conveniently. Thus, we next attempted the intramolecular hydroesterification of alkenylphenols with our catalytic system making use of CO_2_ and PMHS (Table 6). The reaction of 2-vinylphenol (7a) with silyl formate was conducted at 60 °C for 48 h employing dry toluene as solvent, successfully providing lactone 8a in 89% yield (Table 6, entry 1). The different positions of the methyl substituent on the benzene ring of vinylphenols have obvious effects on the reactivity and selectivity of intramolecular hydroesterification reactions. While 4-methyl-2-vinylphenol (7b) and 5-methyl-2-vinylphenol (7c) afforded the five-membered lactones 8b and 8c in good yields, six-membered lactone 8d was obtained in moderate yield from 3-methyl-2-vinylphenol (7d) (Table 6, entries 2–4). For the alkene moiety, the formation of a linear intermediate can favorably alleviate the strong steric hindrance of the *ortho*-methyl group on the benzene ring. To our delight, the electron-withdrawing CO_2_Me group was well tolerated in the *meta*-position, yielding 8e in 78% yield (Table 6, entry 5). When allylphenol 7f was applied to the reaction, the five-membered lactone 8f was furnished in 78% yield (Table 6, entry 6). Besides, 8f can also be constructed from β-methyl-vinylphenol 7g, albeit in lower yield (Table 6, entry 7). Thus, the effectiveness and practicability of our catalytic system employing CO_2_ and PMHS are further demonstrated by the successful implementation of the intramolecular hydroesterification of alkenylphenols.

**Table 6:** The intramolecular hydroesterification of alkenylphenols^a^

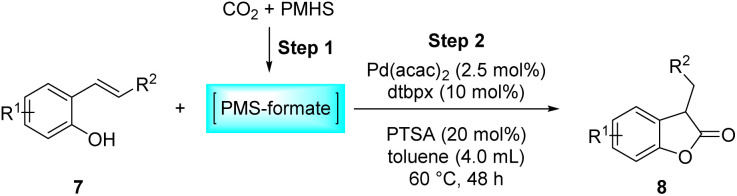			
Entry	Substrate	Product	Yield^b^ (%)
1	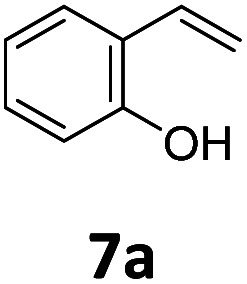	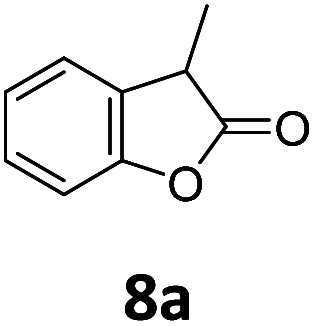	89
2	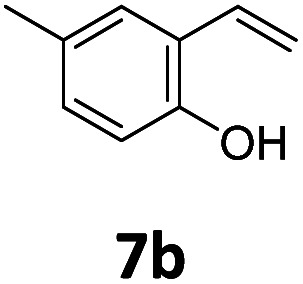	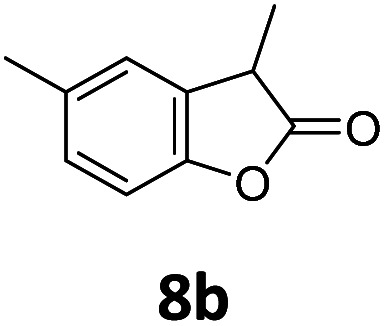	85
3	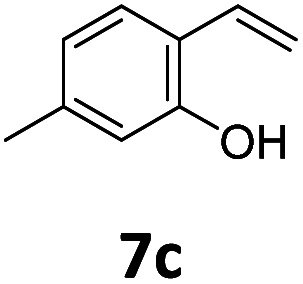	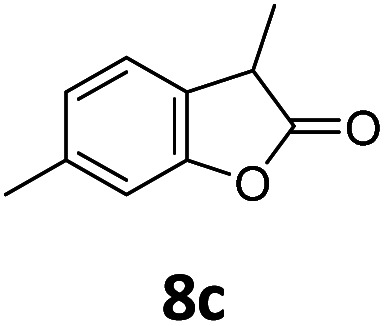	80
4	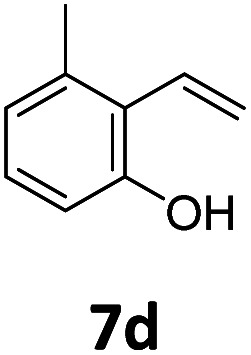	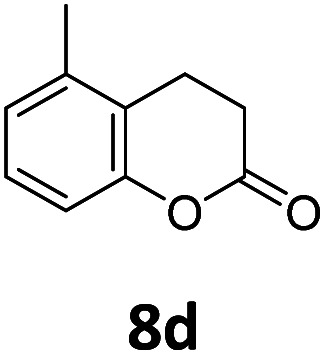	68
5	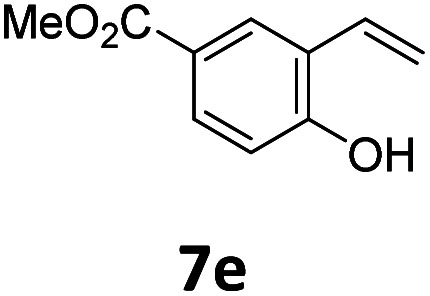	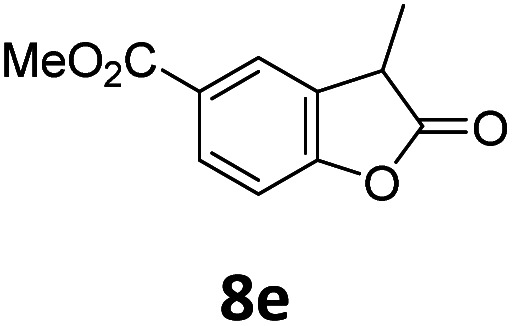	80^c^
6	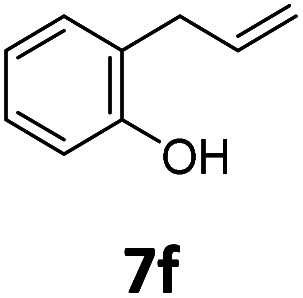	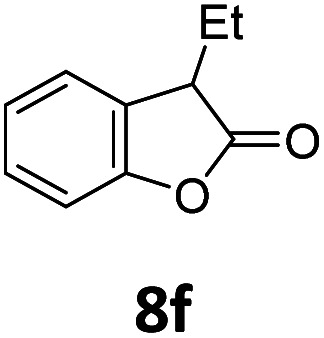	78^d^
7	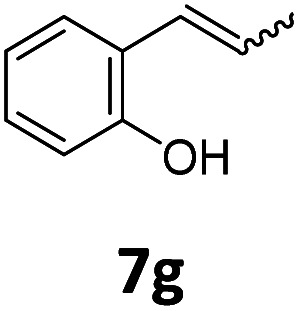	8f	37^c^

a Reaction conditions: step 1: the same as that in Table 1. Step 2: 7 (0.5 mmol), Pd (acac)_2_ (2.5 mol%), dtbpx (10 mol%), PTSA (20 mol%), dry toluene (4.0 mL), 60 °C, 48 h.

b Isolated yields.

c 100 °C, 24 h.

d 100 °C, 16 h.

## Conclusions

In summary, we have successfully implemented palladium-catalyzed hydroesterification of a series of alkenes using CO_2_ and PMHS. Most styrene derivatives perform outstandingly in intermolecular hydroesterification reactions with good to excellent yields and obvious linear or branched selectivity. Moreover, the regioselectivity of intermolecular hydroesterification reactions can also be readily reversed by adjusting reaction parameters such as temperature, solvents and acid additives. Many other types of alkenes are amenable to intermolecular hydroesterification reactions with moderate to nearly quantitative yields and exclusively linear selectivity, including terminal or internal aliphatic alkenes, *N*-derived alkenes, α- or β-methylstyrene, *etc.* Besides, it is noteworthy that intramolecular hydroesterification of alkenylphenols using CO_2_ as a CO source was realized for the first time, obtaining a variety of lactones with important production and medicinal value, which greatly expands the application range of this catalytic system. Further investigation into the catalytic mechanism of this reaction is ongoing.

## Data availability

Additional experimental details and data are provided in the ESI,† including the synthetic procedures for substrates and products and the corresponding NMR and HRMS data.

## Author contributions

M.-M. Wang conceived the project and wrote the manuscript under the supervision of C. Li and S.-M. Lu. All authors discussed the results and contributed to manuscript editing.

## Conflicts of interest

The authors declare no conflict of interest.

## Supplementary Material

SC-014-D3SC01114C-s001
