# Supplementary material for: Regioselective hydroesterification of alkenes and alkenylphenols utilizing CO2 and hydrosilane
Source: Chem Sci. 2023 Apr 18;14(20):5483–9. doi: 10.1039/d3sc01114c (PMC10207877; doi:10.1039/d3sc01114c)
Supplement: SC-014-D3SC01114C-s001 [file SC-014-D3SC01114C-s001.pdf]

Electronic Supplementary Material (ESI) for Chemical Science.
This journal is © The Royal Society of Chemistry 2023

Supplementary Information

Regioselective Hydroesterification of Alkenes and Alkenylphenols Utilizing CO_2 and Hydrosilane

Meng-Meng Wang, ‡[a, b] Sheng-Mei Lu, ‡[a] and Can Li *[a, b]

[a] State Key Laboratory of Catalysis, Dalian Institute of Chemical Physics, Chinese Academy of Sciences, Dalian National Laboratory for Clean Energy, Dalian 116023, China.

[b] University of Chinese Academy of Sciences, Beijing 100049, China.

Corresponding Author: canli@dicp.ac.cn.

Table of Contents

1. General information S2

2. Table S1. List of catalytic systems for hydroesterification of styrene using different CO surrogates S3

3. Synthesis of the Substrates S4

4. Characterization of the Substrates S7

5. Palladium-catalyzed Hydroesterification of Alkenes and Alkenylphenols S10

6. Characterization of the Products S12

7. References S26

8. NMR Spectra S28

1. General Information

All the reagents and solvents were used as received unless otherwise specified. ^{1}H NMR and ^{13}C NMR spectra were recorded in $CDCl_3$ on a Bruker Advance 400 (400 and 100 MHz) NMR and 700 (700 and 175 MHz) NMR spectrometer. Chemical shifts are given in ppm with reference to TMS attributed to 0 ppm and spin-spin coupling constants, *J*, are given in Hz. High resolution mass spectra for all the unknown compounds were done by an LTQ-Orbitrap instrument (ESI) (Thermo Fisher Scientific, USA)

2. Table S1 List of catalytic systems for hydroesterification of styrene using different CO surrogates

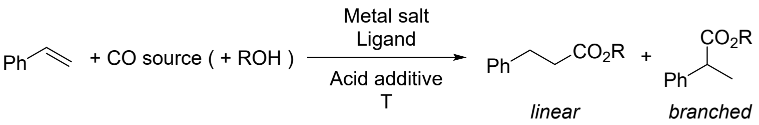

Entry	CO source	Metal salt	Ligand	Acid additive	T/ °C	Yield	*l*:*b*	ref in manuscript
1	CO_2 (balloon)	$Pd(OAc)_2$	dtbpx	PTSA	100	94	3.8:1.0	this work
2	CO_2 (4 MPa)	$Ru_3(CO)_{12}$	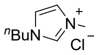 [Bmim]Cl	/	160	no[a]	/	48
3	CO_2 (4 MPa)	$[Ru(CO)_3Cl_2]_2$/ $Co_2(CO)_8$	[Bmin]Cl	/	160	28	2.0:1.0	49
4	HCO_2Bn	$Ru_3(CO)_{12}$	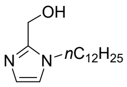	/	135	>99	1.2:1.0	17
5	HCO_2Me	$Pd(acac)_2$	dtbpx	$MeSO_3H$	100	54	8.1:1.0	18
6	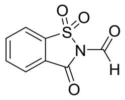 NFS (rt, two chamber)	$Pd(dba)_2$	dtbpx	*rac*-BNPA	rt	80	6.7:1.0	23
7	$(CH_2)O$/MeOH	$Pd(OAc)_2$	dtbpx	PTSA	100	90	3.5:1.0	22
8	HCO_2Ph	$Pd(OAc)_2$	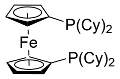	CH_3CO_2H	90	92	>20.0:1.0	19
			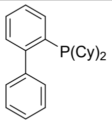	HCO_2H		92	<1.0:20.0	
9	HCO_2Ph	$Pd(OAc)_2$	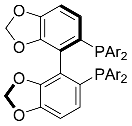 Ar = 3,5-di-*t*Bu-4-MeOPh	/	50	84	18.0:1.0	20
10	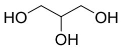 Glycerol (Ir, BQ/160 ºC) (two chamber)	$Pd(dba)_2$	dtbpx	TFA	rt	91	1.0:13.0	16
11	HCO_2H	$Pd(OAc)_2$	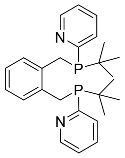	PTSA	100	80	3.0:1.0	21

[a] no = no this substrate. BQ = 1,4-benzoquinone. For entry 1, R = Et; for entries 2, 3, 6, 7, 10 and 11, R = Me.

3. Synthesis of the Substrates

Synthesis of alkenes 1 and alkenylphenols 7

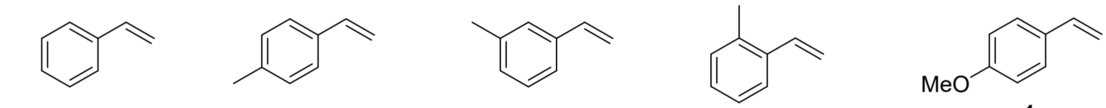

1a **1b** **1c** **1d** **1e**

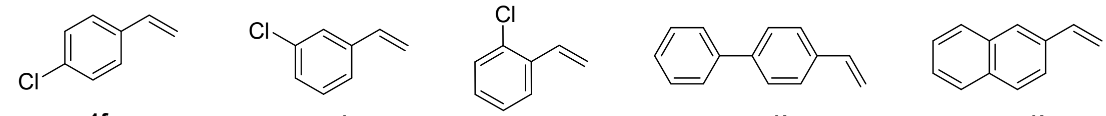

1f **1g** **1h** **1i** **1j**

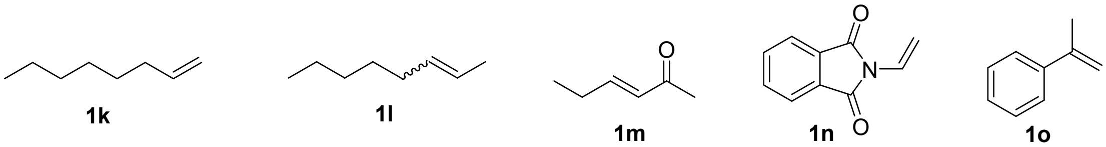

1k **1l** **1m** **1n** **1o**

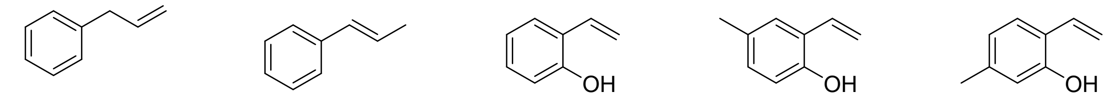

1p **1q** **7a** **7b** **7c**

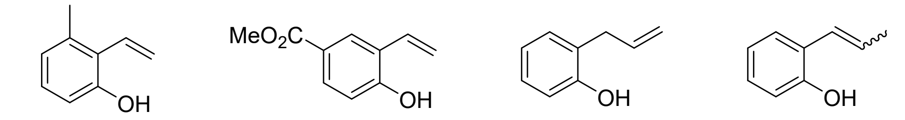

7d **7e** **7f** **7g**

Compounds **1a-1m**, **1o-1q** and **7f** were purchased from the chemical regent company. Compound **1n**[1], **7a-7e**[2] and **7g**[2] were prepared according to the literature procedures. The preparation of new compounds and their characterization data are provided as follows.

Synthesis of 1n

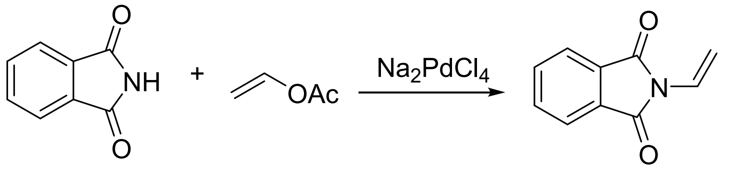

1n

Typical procedure A for the preparation of 1n[1]: To a stirred solution of phthalimide (0.88 g, 6.0 mmol) in vinyl acetate (15 mL, 26.8 equiv.) under argon was

added Na_2PdCl_4 (17.6 mg) and the mixture was heated under reflux for 8 h. Activated charcoal (15 mg) was then added and the mixture was stirred for 10 min. It was then diluted with diethyl ether (20 mL), the solids were removed by filtration, and the filtrate was concentrated to dryness under high vacuum. The crude product was extracted with diethyl ether and the combined extracts were dried over $MgSO_4$ and concentrated in vacuo. The residue was purified by flash column chromatography (10:1 hexane/EtOAc) to afford the product.

Synthesis of substrates 7a-7e and 7g

Wittig reactions

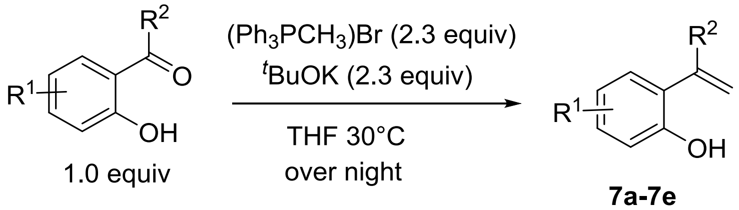

7a-7e

General procedure B for the Witting reactions[2]: A flame-dried Schlenk tube was charged with methyltriphenylphosphonium bromide (8.22g, 23.0 mmol) and potassium tert-butoxide (2.58g, 23.0 mmol), which were suspended in anhydrous THF. A solution of the salicylaldehyde (10.0 mmol, 1.0 equiv) in anhydrous THF was added to the orange suspension via syringe. The reaction mixture was heated up to 30 °C and stirred overnight. The reaction mixture was quenched with saturated aqueous ammonium chloride solution and extracted with Et_2O. The combined organic layers were washed with brine, dried over $MgSO_4$ and the solvent was removed under reduced pressure. The crude product was purified by column chromatography.

Synthesis of β-methyl-vinylphenol

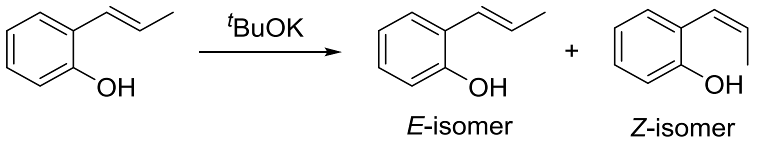

(*E*)-2-(Prop-1-en-1-yl)phenol and (*Z*)-2-(prop-1-en-1-yl)phenol (**7g**)

Typical procedure C for the preparation of 7g[2]: A flame-dried Schlenk tube was charged with potassium tert-butoxide (4.50 g, 40.0 mmol, 4.0 equiv), which was dissolved in anhydrous THF. Allylphenol (1.30 mL, 10.0 mmol, 1.0 equiv) was added via syringe and the solution was stirred at ambient temperature overnight. The reaction mixture was quenched with saturated aqueous ammonium chloride solution and extracted with Et_2O (3 x 20 mL). The combined organic layers were washed with brine, dried over $MgSO_4$ and the solvent was removed under reduced pressure. The crude product was purified by column chromatography.

4. Characterization of the Substrates

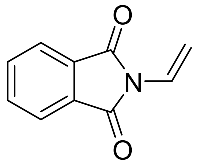

1n

N-Vinylphthalimide (**1n**): The title compound was prepared by following the typical procedure A as a white solid in 97% yield. The obtained analytical data are in accordance to the literature.[1]

^{1}H NMR (400 MHz, $CDCl_3$) δ 7.95-7.65 (m, 4H), 6.88 (dd, *J* = 16.4, 9.9 Hz, 1H), 6.09 (d, *J* = 16.4 Hz, 1H), 5.05 (d, *J* = 9.9 Hz, 1H). ^{13}C NMR (100 MHz, $CDCl_3$) δ 166.5, 134.5, 131.6, 123.8, 123.7, 104.5.

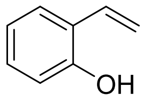

7a

2-Vinylphenol (**7a**): The title compound was prepared by following the general procedure B as a light yellowish white solid in 95% yield. The obtained analytical data are in accordance to the literature.[2]

^{1}H NMR (400 MHz, $CDCl_3$) δ 7.38 (dd, *J* = 7.7, 1.4 Hz, 1H), 7.14 (td, *J* = 7.8, 1.6 Hz, 1H), 7.01-6.87 (m, 2H), 6.79 (d, *J* = 8.1 Hz, 1H), 5.74 (dd, *J* = 17.7, 1.2 Hz, 1H), 5.36 (dd, *J* = 11.2, 1.2 Hz, 1H), 4.98 (s, 1H). ^{13}C NMR (100 MHz, $CDCl_3$) δ 152.7, 131.5, 128.9, 127.4, 124.8, 121.0, 115.9, 115.8.

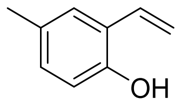

7b

4-Methyl-2-vinylphenol (**7b**): The title compound was prepared by following the general procedure B as a light yellowish white solid in 83% yield. The obtained analytical data are in accordance to the literature.[3,4]

^{1}H NMR (400 MHz, $CDCl_3$) δ 7.18 (s, 1H), 7.03-6.82 (m, 2H), 6.68 (d, *J* = 8.1 Hz, 1H), 5.72 (d, *J* = 17.7 Hz, 1H), 5.33 (d, *J* = 11.2 Hz, 1H), 2.27 (s, 3H). ^{13}C NMR (100 MHz, $CDCl_3$) δ 150.7, 131.6, 130.1, 129.4, 127.7, 124.5, 115.8, 115.5, 20.5.

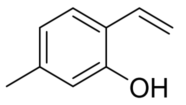

7c

5-Methyl-2-vinylphenol (**7c**): The title compound was prepared by following the general procedure B as a white solid in 82% yield. The obtained analytical data are in accordance to the literature.[2]

^{1}H NMR (700 MHz, $CDCl_3$) δ 7.26 (d, *J* = 7.8 Hz, 1H), 6.89 (dd, *J* = 17.7, 11.2 Hz, 1H), 6.73 (d, *J* = 7.8 Hz, 1H), 6.61 (s, 1H), 5.69 (dd, *J* = 17.8, 0.9 Hz, 1H), 5.30 (dd, *J* = 11.2, 0.9 Hz, 1H), 4.93 (s, 1H), 2.29 (s, 3H). ^{13}C NMR (175 MHz, $CDCl_3$) δ 152.6, 139.2, 131.4, 127.2, 122.0, 121.8, 116.5, 114.9, 21.1.

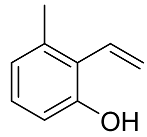

7d

3-Methyl-2-vinylphenol (**7d**): The title compound was prepared by following the general procedure B as a light yellowish oil, which solidified in the fridge (70% yield). The obtained analytical data are in accordance to the literature.[5]

^{1}H NMR (400 MHz, $CDCl_3$) δ 7.03 (t, *J* = 7.8 Hz, 1H), 6.74 (dd, *J* = 13.2, 7.9 Hz, 2H), 6.66 (dd, *J* = 18.2, 11.7 Hz, 1H), 5.77-5.43 (m, 3H), 2.24 (s, 3H). ^{13}C NMR (100 MHz, $CDCl_3$) δ 152.8, 137.3, 132.3, 128.3, 124.0, 122.0, 120.5, 113.1, 20.2. HRMS (ESI+, m/z): calculated for $C_9H_{11}O$ $[M+H]^+$ 135.0810; found 135.0806.

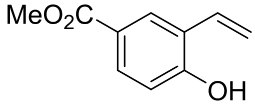

7e

Methyl 4-hydroxy-3-vinylbenzoate (**7e**): The title compound was prepared by following the general procedure B as a light yellowish solid in 78% yield. The obtained analytical data are in accordance to the literature.[2,4,6]

^{1}H NMR (400 MHz, $CDCl_3$) δ 8.13 (d, *J* = 1.5 Hz, 1H), 7.82 (dd, *J* = 8.4, 1.9 Hz, 1H), 7.16 (d, *J* = 2.4 Hz, 1H), 6.97 (dd, *J* = 17.7, 11.2 Hz, 1H), 6.89 (d, *J* = 8.5 Hz, 1H), 5.82 (d, *J* = 17.7 Hz, 1H), 5.37 (d, *J* = 11.2 Hz, 1H), 3.92 (s, 3H). ^{13}C NMR (100 MHz, $CDCl_3$) δ 167.9, 157.8, 130.7, 130.7, 129.3, 125.0, 122.0, 116.5, 115.8, 52.3.

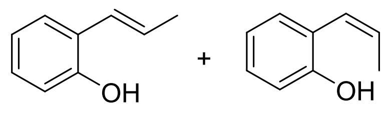

7g

E-isomer *Z*-isomer

(*E*)-2-(Prop-1-en-1-yl)phenol and (*Z*)-2-(prop-1-en-1-yl)phenol (**7g**): The title compound was prepared by following the typical procedure C as a colorless oil in 90% yield (*E*/*Z* = 77/23). The obtained analytical data are in accordance to the literature.[2]

E-isomer：

^{1}H NMR (400 MHz, $CDCl_3$) δ 7.29 (dd, *J* = 7.7, 1.5 Hz, 1H), 7.14-7.04 (m, 1H), 6.95-6.84 (m, 1H), 6.78 (dd, *J* = 8.0, 1.0 Hz, 1H), 6.58 (dd, *J* = 15.9, 1.6 Hz, 1H), 6.20 (dq, *J* = 15.8, 6.6 Hz, 1H), 5.05 (s, 1H), 1.91 (dd, *J* = 6.6, 1.7 Hz, 3H). ^{13}C NMR (100 MHz, $CDCl_3$) δ 152.3, 128.4, 128.0, 127.4, 125.3, 124.0, 120.9, 115.7, 19.0.

Z-isomer：

^{1}H NMR (400 MHz, $CDCl_3$) δ 7.21-7.15 (m, 1H), 7.14-7.04 (m, 1H), 6.95-6.84 (m, 2H), 6.39 (dt, *J* = 10.4, 5.2 Hz, 1H), 6.02 (dq, *J* = 11.2, 7.0 Hz, 1H), 5.28 (s, 1H), 1.72 (dd, *J* = 7.0, 1.8 Hz, 3H). ^{13}C NMR (100 MHz, $CDCl_3$) δ 152.6, 131.3, 129.7, 128.6, 125.1, 123.5, 120.3, 115.1, 14.6.

5. Palladium-catalyzed Hydroesterification of Alkenes and Alkenylphenols

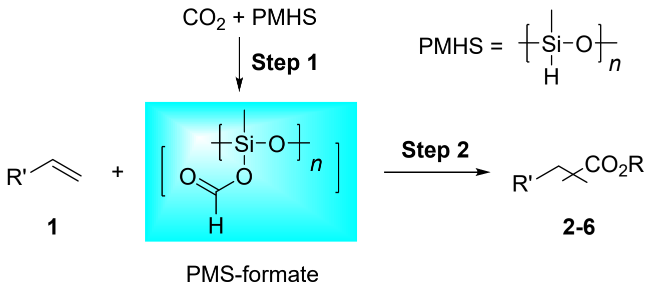

General procedure I for the intermolecular hydroesterification of alkenes utilizing CO_2 and PMHS: A 35 mL Schlenk tube equipped with a magnetic stir bar was fitted with a balloon filled with CO_2. The tube was charged with $Cu(OAc)_2$ (0.01 mmol, 1.0 mol%), dppbz (0.015 mmol, 1.5 mol%), and a solution of PMHS (Acros, *FW*: ca. 1900, 0.165 g, Si-H, 2.5 mmol, 2.5 equiv) in dry 1,4-dioxane (0.5 mL). The tube was then flushed with CO_2. After that, the tube was tightly sealed under the balloon pressure of CO_2 and stirred at 65 °C for 30 min. A yellow solution was obtained. Once completed, the tube containing the silyl formate solution was removed from the oil bath. Then, dry ROH (MeOH, EtOH, ^{n}PrOH, ^{i}PrOH or ^{t}BuOH, 4 mL), **1** (1.0 mmol, 1.0 equiv), $Pd(acac)_2$ (2.5 mol%) or $Pd(OAc)_2$ (2.5 mol%), dtbpx (10 mol%), PTSA (20 mol%) were added sequentially under CO_2 atmosphere. After that, CO_2 balloon was removed, and the Schlenk tube was tightly sealed and stirred at 100 °C for 16-24 h or 60 °C for 48 h. After the reaction mixture was cooled to room temperature, the solvent was concentrated in vacuo. Purification of the residue by flash column chromatography to afford the ester products **2-6**.

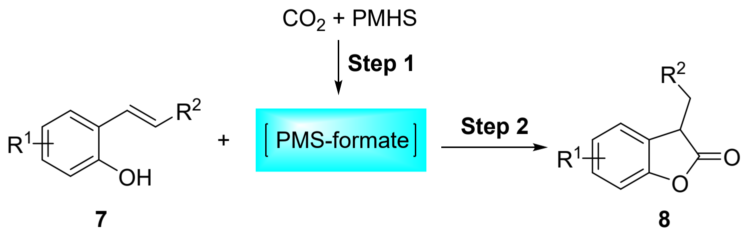

General procedure II for the intramolecular hydroesterification of alkenylphenols utilizing CO_2 and PMHS: A 35 mL Schlenk tube equipped with a magnetic stir bar was fitted with a balloon filled with CO_2. The tube was charged with $Cu(OAc)_2$ (0.005 mmol, 1.0 mol%), dppbz (0.0075 mmol, 1.5 mol%), and a solution of PMHS (Acros, *FW*: ca. 1900, 0.083 g, Si-H, 1.25 mmol, 2.5 equiv) in dry 1,4-dioxane (0.5 mL). The tube was then flushed with CO_2. After that, the tube was tightly sealed at balloon pressure of CO_2, and stirred at 65 °C for 30 min, resulting in a yellow solution. Once completed, the tube containing the silyl formate solution was removed from the oil bath. Then, dry toluene (4 mL), alkenylphenol **7** (0.5 mmol, 1.0 equiv), $Pd(acac)_2$ (2.5 mol%), dtbpx (10 mol%) and PTSA (20 mol%) were added sequentially under CO_2 atmosphere. After that, CO_2 balloon was removed, and the Schlenk tube was tightly sealed and stirred at 60 °C for 48 h or 100 °C for 16-24 h. After the reaction mixture was cooled to room temperature, the solvent was concentrated in vacuo. Purification of the residue by flash column chromatography to afford the lactone products **8**.

6. Characterization of the Products

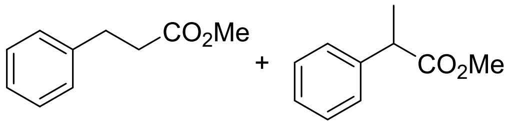

2a-*l* **2a-*b***

Methyl 3-phenylpropanoate (2a-*l*) and methyl 3-phenylpropanoate (2a-*b*) (Table 1, entry 1): From styrene (**1a**) (104.2 mg, 1.0 mmol), the title compound was prepared by following the general procedure I (100 °C, 16 h, $Pd(OAc)_2$, MeOH) as a yellow oil (151.1 mg, 92% yield, *l*/*b* = 2.7/1.0). The spectroscopic data correspond to those previously reported in the literature.

2a-*l*:[7,8]

^{1}H NMR (400 MHz, $CDCl_3$) δ 7.43-7.11 (m, 5H), 3.67 (s, 3H), 2.95 (t, *J* = 7.9 Hz, 2H), 2.63 (t, *J* = 7.9 Hz, 2H). ^{13}C NMR (100 MHz, $CDCl_3$) δ 173.4, 140.5, 128.5, 128.3, 126.3, 51.6, 35.7, 31.0.

2a-*b*:[9-11]

^{1}H NMR (400 MHz, $CDCl_3$) δ 7.43-7.11 (m, 5H), 3.72 (q, *J* = 7.2 Hz, 1H), 3.66 (s, 3H), 1.50 (d, *J* = 7.2 Hz, 3H). ^{13}C NMR (100 MHz, $CDCl_3$) δ 175.0, 140.6, 128.6, 127.5, 127.2, 52.0, 45.4, 18.6.

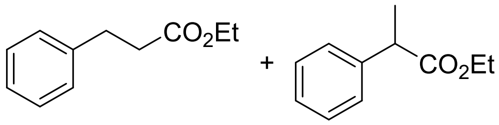

3a-*l* **3a-*b***

Ethyl 3-phenylpropanoate (3a-*l*) and ethyl 2-phenylpropanoate (3a-*b*) (Table 1, entry 2): From styrene (**1a**) (104.2 mg, 1.0 mmol), the title compound was prepared by following the general procedure I (100 °C, 16 h, $Pd(OAc)_2$, EtOH) as a yellow oil (167.5 mg, 94% yield, *l*/*b* = 3.8/1.0). The spectroscopic data correspond to those previously reported in the literature.

3a-*l*:[7,12,13]

^1H NMR (400 MHz, $CDCl_3$) δ 7.35-7.16 (m, 5H), 4.12 (q, J = 7.2 Hz, 2H), 2.95 (t, J = 7.8 Hz, 2H), 2.62 (t, J = 7.8 Hz, 2H), 1.23 (t, J = 7.1 Hz, 3H). ^{13}C NMR (100 MHz, $CDCl_3$) δ 172.9, 140.6, 128.5, 128.3, 126.2, 60.4, 36.0, 31.0, 14.2.

3a-*b*:[10,14,15]

^1H NMR (400 MHz, $CDCl_3$) δ 7.35-7.15 (m, 5H), 4.20-4.05 (m, 2H), 3.70 (q, J = 7.2 Hz, 1H), 1.49 (d, J = 7.2 Hz, 3H), 1.22-1.17 (m, 3H). ^{13}C NMR (100 MHz, $CDCl_3$) δ 174.6, 140.7, 128.6, 127.5, 127.1, 60.7, 45.6, 18.6, 14.1.

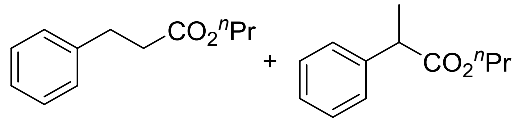

4a-*l*   **4a-*b***

Propyl 3-phenylpropanoate (4a-*l*) and propyl 2-phenylpropanoate (4a-*b*) (Table 1, entry 3): From styrene (**1a**) (104.2 mg, 1.0 mmol), the title compound was prepared by following the general procedure I (100 °C, 16 h, $Pd(OAc)_2$, ^{n}PrOH) as a yellow oil (96.1 mg, 50% yield, *l*/*b* = 2.3/1.0). The spectroscopic data correspond to those previously reported in the literature.

4a-*l*:[16]

^1H NMR (400 MHz, $CDCl_3$) δ 7.35-7.16 (m, 5H), 4.03 (t, J = 6.7 Hz, 2H), 3.02-2.89 (m, 2H), 2.69-2.58 (m, 2H), 1.69-1.55 (m, 2H), 0.91 (t, J = 7.4 Hz, 3H). ^{13}C NMR (100 MHz, $CDCl_3$) δ 173.1, 140.6, 128.5, 128.3, 126.2, 66.1, 36.0, 31.0, 22.0, 10.4.

4a-*b*:[17]

^1H NMR (400 MHz, $CDCl_3$) δ 7.38-7.14 (m, 5H), 4.02 (t, J = 6.7 Hz, 2H), 3.72 (q, J = 7.2 Hz, 1H), 1.64-1.53 (m, 2H), 1.50 (d, J = 7.2 Hz, 3H), 0.85 (t, J = 7.4 Hz, 3H). ^{13}C NMR (100 MHz, $CDCl_3$) δ 174.6, 140.7, 128.6, 127.5, 127.1, 66.3, 45.6, 21.9, 18.5, 10.3.

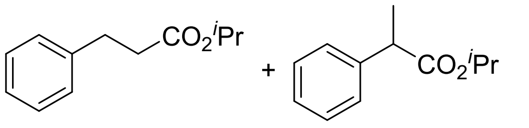

5a-*l* **5a-*b***

Isopropyl 3-phenylpropanoate (5a-*l*) and isopropyl 2-phenylpropanoate (5a-*b*) (Table 1, entry 4)：From styrene (**1a**) (104.2 mg, 1.0 mmol), the title compound was prepared by following the general procedure I (100 °C, 16 h, $Pd(OAc)_2$, $^{i}PrOH$) as a yellow oil (38.4 mg, 20% yield, *l*/*b* = 4.5/1.0). The spectroscopic data correspond to those previously reported in the literature.

5a-*l*:[7,10,18]

^{1}H NMR (400 MHz, $CDCl_3$) δ 7.33-7.24 (m, 2H), 7.24-7.16 (m, 3H), 5.00 (dt, *J* = 12.5, 6.3 Hz, 1H), 2.99-2.89 (m, 2H), 2.63-2.55 (m, 2H), 1.20 (d, *J* = 6.3 Hz, 2H). ^{13}C NMR (100 MHz, $CDCl_3$) δ 172.5, 140.6, 128.5, 128.3, 126.2, 67.7, 36.3, 31.1, 21.8.

5a-*b*:[10]

^{1}H NMR (400 MHz, $CDCl_3$) δ 7.37-7.15 (m, 5H), 5.06-4.89 (m, 1H), 3.67 (q, *J* = 7.2 Hz, 1H), 1.48 (d, *J* = 7.2 Hz, 3H), 1.23-1.18 (m, 3H), 1.13 (d, *J* = 6.2 Hz, 3H). ^{13}C NMR (100 MHz, $CDCl_3$) δ 58.5, 18.4.

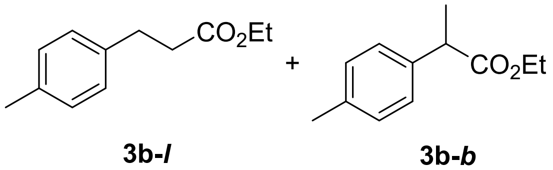

3b-*l* **3b-*b***

Ethyl 3-(p-tolyl)propanoate (3b-*l*) and ethyl 2-(p-tolyl)propanoate (3b-*b*) (Table 4, entry 2): From 4-methylstyrene (**1b**) (118.2 mg, 1.0 mmol), the title compound was prepared by following the general procedure I (100 °C, 16 h, $Pd(OAc)_2$, EtOH) as a yellow oil (174.9 mg, 91% yield, *l*/*b* = 4.3/1.0). The spectroscopic data correspond to those previously reported in the literature.

3b-*l*:[19]

^{1}H NMR (400 MHz, $CDCl_3$) δ 7.23-6.90 (m, 4H), 4.12 (q, J = 7.1 Hz, 2H), 2.91 (t, J = 7.8 Hz, 2H), 2.59 (t, J = 7.8 Hz, 2H), 2.31 (s, 3H), 1.24 (t, J = 7.0 Hz, 3H). ^{13}C NMR (100 MHz, $CDCl_3$) δ 173.0, 137.5, 135.7, 129.2, 128.2, 60.4, 36.1, 30.6, 21.0, 14.2.

3b-*b*:[15,20]

^{1}H NMR (400 MHz, $CDCl_3$) δ 7.22-6.93 (m, 4H), 4.18-4.03 (m, 2H), 3.66 (q, J = 7.2 Hz, 1H), 2.32 (s, 3H), 1.47 (d, J = 7.1 Hz, 2H), 1.23-1.17 (m, 3H). ^{13}C NMR (100 MHz, $CDCl_3$) δ 129.3, 127.3, 60.7, 45.2, 21.0, 18.7, 14.1.

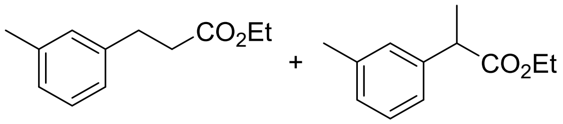

3c-*l*　　**3c-*b***

Ethyl 3-(*m*-tolyl)propanoate (3c-*l*) and ethyl 2-(*m*-tolyl)propanoate (3c-*b*) (Table 4, entry 3): From 3-methylstyrene (**1c**) (118.2 mg, 1.0 mmol), the title compound was prepared by following the general procedure I (100 °C, 16 h, $Pd(OAc)_2$, EtOH) as a yellow oil (169.2 mg, 88% yield, *l*/*b* = 3.8/1.0). The spectroscopic data correspond to those previously reported in the literature.

3c-*l*:[21]

^{1}H NMR (400 MHz, $CDCl_3$) δ 7.30-6.93 (m, 4H), 4.22-4.03 (m, 2H), 2.91 (t, J = 7.9 Hz, 2H), 2.60 (t, J = 7.9 Hz, 2H), 2.32 (s, 3H), 1.23 (t, J = 7.1 Hz, 3H). ^{13}C NMR (100 MHz, $CDCl_3$) δ 173.0, 140.6, 138.0, 129.1, 128.4, 127.0, 125.3, 60.4, 36.0, 30.9, 21.4, 14.2.

3c-*b*:[22-24]

^{1}H NMR (400 MHz, $CDCl_3$) δ 7.27-6.96 (m, 4H), 4.20-4.03 (m, 2H), 3.66 (q, J = 7.1 Hz, 1H), 2.34 (s, 3H), 1.48 (d, J = 7.1 Hz, 3H), 1.21 (t, J = 7.1 Hz, 3H). ^{13}C NMR (100 MHz, $CDCl_3$) δ 174.7, 140.7, 138.2, 128.5, 128.2, 127.8, 124.5, 60.7, 45.5, 21.4, 18.7, 14.1.

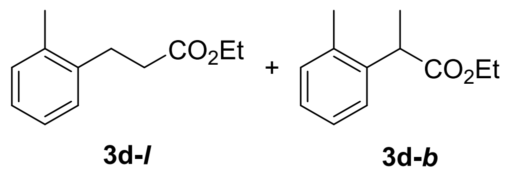

Ethyl 3-(*o*-tolyl)propanoate (3d-*l*) and ethyl 2-(*o*-tolyl)propanoate (3d-*b*) (Table 4, entry 4)**:** From 2-methylstyrene (**1d**) (118.2 mg, 1.0 mmol), the title compound was prepared by following the general procedure I (100 °C, 16 h, $Pd(OAc)_2$, EtOH) as a yellow oil (178.0 mg, 93% yield, *l*/*b* = 10.1/1.0). The spectroscopic data correspond to those previously reported in the literature.

3d-*l*:[25]

^{1}H NMR (400 MHz, $CDCl_3$) δ 7.19-7.07 (m, 4H), 4.14 (q, *J* = 7.1 Hz, 2H), 2.99-2.90 (m, 2H), 2.64-2.53 (m, 2H), 2.33 (s, 3H), 1.25 (t, *J* = 7.1 Hz, 3H). ^{13}C NMR (100 MHz, $CDCl_3$) δ 173.1, 138.7, 136.0, 130.3, 128.5, 126.4, 126.1, 60.5, 34.7, 28.4, 19.3, 14.2.

3d-*b*:[22,23]

^{1}H NMR (400 MHz, $CDCl_3$) δ 7.21-7.03 (m, 4H), 4.14-4.03 (m, 2H), 3.94 (dd, *J* = 14.4, 7.2 Hz, 1H), 2.37 (s, 3H), 1.46 (d, *J* = 7.1 Hz, 3H), 1.20 (t, *J* = 7.2 Hz, 3H).

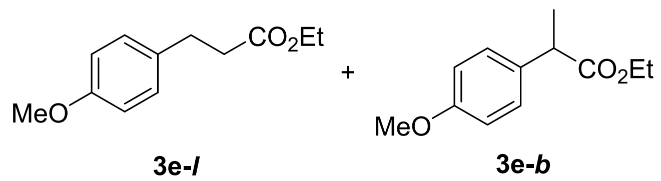

Ethyl 3-(4-methoxyphenyl)propanoate (3e-*l*) and ethyl 2-(4-methoxyphenyl)propanoate (3e-*b*) (Table 4, entry 5): From 4-methoxystyrene (**1e**) (134.2 mg, 1.0 mmol), the title compound was prepared by following the general procedure I (100 °C, 16 h, $Pd(OAc)_2$, EtOH) as a yellow oil (83.3 mg, 35% yield, *l*/*b* = 4.9/1.0). The spectroscopic data correspond to those previously reported in the literature.

3e-*l*:[7,13]

^{1}H NMR (400 MHz, $CDCl_3$) δ 7.12 (d, J = 8.2 Hz, 2H), 6.83 (d, J = 8.2 Hz, 2H), 4.12 (q, J = 7.1 Hz, 2H), 3.78(s, 3H), 2.89 (t, J = 7.8 Hz, 2H), 2.58 (t, J = 7.8 Hz, 2H), 1.23 (t, J = 7.1 Hz, 3H). ^{13}C NMR (100 MHz, $CDCl_3$) δ 173.0, 158.1, 132.7, 129.3, 113.9, 60.4, 55.3, 36.3, 30.1, 14.2.

3e-*b*:[23,24,26]

^{1}H NMR (400 MHz, $CDCl_3$) δ 7.23 (d, J = 8.3 Hz, 2H), 6.90-6.80 (m, 2H), 4.12 (q, J = 7.1 Hz, 2H), 3.78(s, 3H), 3.65 (q, J = 7.1 Hz, 1H), 1.47 (d, J = 7.1 Hz, 3H), 1.23-1.17 (m, 3H). ^{13}C NMR (100 MHz, $CDCl_3$) δ 174.8, 158.6, 132.8, 128.5, 114.0, 60.7, 55.3, 44.7, 18.7, 14.2.

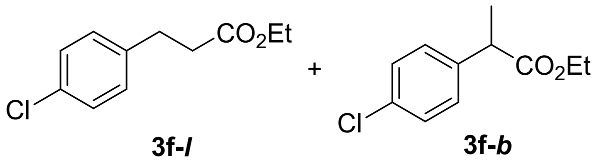

Ethyl 3-(4-chlorophenyl)propanoate (3f-*l*) and ethyl 2-(4-chlorophenyl)propanoate (3f-*b*) (Table 4, entry 6): From 4-chlorostyrene (**1f**) (138.6 mg, 1.0 mmol), the title compound was prepared by following the general procedure I (60 °C, 48 h, $Pd(OAc)_2$, EtOH) as a yellow oil (168.0 mg, 79% yield, *l*/*b* = 1.0/2.1). The spectroscopic data correspond to those previously reported in the literature.

3f-*l*:[27-29]

^{1}H NMR (400 MHz, $CDCl_3$) δ 7.33-7.19 (m, 2H), 7.13 (d, J = 8.0 Hz, 2H), 4.22-4.02 (m, 2H), 2.92 (t, J = 7.7 Hz, 2H), 2.59 (t, J = 7.7 Hz, 2H), 1.30-1.20(m, 3H). ^{13}C NMR (100 MHz, $CDCl_3$) δ 172.6, 139.0, 132.0, 129.7, 128.6, 60.5, 35.8, 30.3, 14.2.

3f-*b*:[23]

^{1}H NMR (400 MHz, $CDCl_3$) δ 7.33-7.19 (m, 4H), 4.22-4.00 (m, 2H), 3.68 (q, J = 7.1 Hz, 1H), 1.47 (d, J = 7.2 Hz, 2H), 1.20 (t, J = 7.2 Hz, 3H). ^{13}C NMR (100 MHz, $CDCl_3$) δ 174.1, 139.1, 132.9, 128.9, 128.7, 60.9, 45.0, 18.5, 14.1.

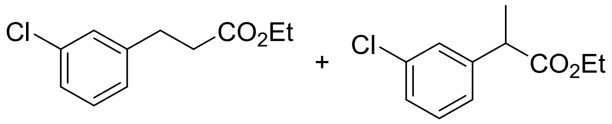

3g-*l* **3g-*b***

Ethyl 3-(3-chlorophenyl)propanoate (3g-*l*) and ethyl 2-(3-chlorophenyl)propanoate (3g-*b*) (Table 4, entry 7): From 3-chlorostyrene (**1g**) (138.6 mg, 1.0 mmol), the title compound was prepared by following the general procedure I (60 °C, 48 h, $Pd(acac)_2$, EtOH) as a yellow oil (178.6 mg, 84% yield, *l*/*b* = 1.0/2.1). The spectroscopic data correspond to those previously reported in the literature.

3g-*l*:[30]
^{1}H NMR (400 MHz, $CDCl_3$) δ 7.34-7.04 (m, 4H), 4.28-4.08 (m, 2H), 2.93 (t, *J* = 7.7 Hz, 2H), 2.61 (t, *J* = 7.7 Hz, 2H), 1.21 (t, *J* = 7.2 Hz, 3H). ^{13}C NMR (100 MHz, $CDCl_3$) δ 172.5, 142.6, 134.2, 129.7, 128.5, 126.6, 126.5, 60.5, 35.6, 30.6, 14.2.

3g-*b*:[22]
^{1}H NMR (400 MHz, $CDCl_3$) δ 7.34-7.16 (m, 4H), 4.28-4.08 (m, 2H), 3.67 (q, *J* = 7.2 Hz, 1H), 1.48 (d, *J* = 7.2 Hz, 3H), 1.23 (t, *J*= 7.2 Hz, 3H). ^{13}C NMR (100 MHz, $CDCl_3$) δ 173.9, 142.6, 134.4, 129.8, 127.8, 127.3, 125.7, 61.0, 45.3, 18.5, 14.1.

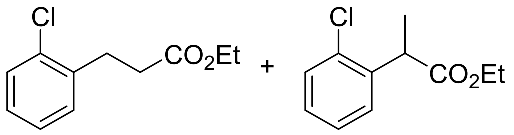

3h-*l* **3h-*b***

Ethyl 3-(2-chlorophenyl)propanoate (3h-*l*) and ethyl 2-(2-chlorophenyl)propanoate (3h-*b*) (Table 4, entry 8): From 2-chlorostyrene (**1h**) (138.6 mg, 1.0 mmol), the title compound was prepared by following the general procedure I (60 °C, 48 h, $Pd(acac)_2$, EtOH) as a yellow oil (172.3 mg, 81% yield, *l*/*b* = 4.9/1.0). The spectroscopic data correspond to those previously reported in the literature.

For **3h-*l***:[30]

^1H NMR (400 MHz, $CDCl_3$) ^1H NMR (400 MHz, $CDCl_3$) δ 7.39-7.29 (m, 1H), 7.28-7.21 (m, 1H), 7.21-7.10 (m, 2H), 4.13 (q, *J* = 7.1 Hz, 2H), 3.08-3.03 (m, 1H), 2.66-2.61 (m, 1H), 1.23 (t, *J* = 7.2 Hz, 3H). ^{13}C NMR (100 MHz, $CDCl_3$) δ 172.7, 138.1, 134.0, 130.5, 129.6, 127.8, 126.9, 60.5, 34.0, 29.0, 14.2.

Minor signals from **3h-*b***:

^1H NMR (400 MHz, $CDCl_3$) ^1H NMR (400 MHz, $CDCl_3$) δ 7.38-7.12 (m, 4H), 4.23-4.16 (m, 1H), 4.16-4.10 (m, 2H), 1.49 (d, *J* = 7.2 Hz, 3H), 1.21 (t, *J* = 7.1 Hz, 3H). ^{13}C NMR (100 MHz, $CDCl_3$) δ 174.0, 138.6, 133.7, 129.6, 128.4, 128.2, 127.1, 60.9, 42.2, 17.6, 14.1.

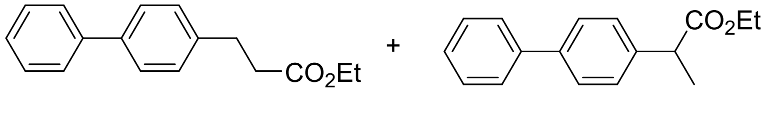

3i-*l* **3i-*b***

Ethyl 3-([1,1'-biphenyl]-4-yl)propanoate (3i-*l*) and ethyl 2-([1,1'-biphenyl]-4-yl)propanoate (3i-*b*) (Table 4, entry 9): From 4-vinylbiphenyl (**1i**) (180.2 mg, 1.0 mmol), the title compound was prepared by following the general procedure I (100 °C, 24 h, $Pd(OAc)_2$, EtOH) as a yellow oil (203.4 mg, 80% yield, *l*/*b* = 2.3/1.0). The spectroscopic data correspond to those previously reported in the literature.

For **3i-*l***:[30,31]

^1H NMR (400 MHz, $CDCl_3$) δ 7.54 (dd, *J* = 21.2, 7.7 Hz, 4H), 7.42 (t, *J* = 7.4 Hz, 2H), 7.35-7.22 (m, 3H), 4.14 (q, *J* = 7.1 Hz, 2H), 2.99 (t, *J* = 7.8 Hz, 2H), 2.66 (t, *J* = 7.8 Hz, 2H), 1.24 (t, *J* = 7.0 Hz, 3H). ^{13}C NMR (100 MHz, $CDCl_3$) δ 172.9, 141.0, 139.7, 139.2, 128.8, 128.8, 127.2, 127.1, 127.0, 60.5, 35.9, 30.6, 14.2.

minor signals from **3i-*b***:[24]

^1H NMR (400 MHz, $CDCl_3$) δ 7.64-7.20 (m, 9H), 4.22-4.08 (m, 2H), 3.75 (q, *J* = 7.1 Hz, 1H), 1.53 (d, *J* = 7.1 Hz, 3H), 1.29-1.20 (m, 3H). ^{13}C NMR (100 MHz, $CDCl_3$) δ 174.6, 140.8, 140.0, 139.8, 128.5, 127.9, 127.4, 127.3, 127.1, 60.8, 45.3, 18.7, 14.2.

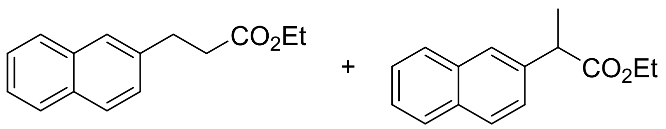

3j-*l* **3j-*b***

Ethyl 2-(naphthalen-2-yl)propanoate (3j-*l*) and ethyl 3-(naphthalen-2-yl)propanoate (3j-*b*) (Table 4, entry 10): From 2-vinylnaphthalene (**1j**) (154.2 mg, 1.0 mmol), the title compound was prepared by following the general procedure I (100 °C, 24 h, Pd(acac)$_2$) as a yellow oil (153.0 mg, 67% yield, *l*/*b* = 2.8/1.0). The spectroscopic data correspond to those previously reported in the literature.

For **3j-*l***:[30]

^{1}H NMR (400 MHz, CDCl$_3$) δ 7.86-7.76 (m, 3H), 7.67 (s, 1H), 7.52-7.42 (m, 2H), 7.37 (dd, *J* = 8.4, 1.3 Hz, 1H), 4.16 (q, *J* = 7.1 Hz, 2H), 3.14 (t, *J* = 7.8 Hz, 2H), 2.74 (t, *J* = 7.8 Hz, 2H), 1.26 (t, *J* = 7.1 Hz, 3H). ^{13}C NMR (100 MHz, CDCl$_3$) δ 172.9, 138.1, 133.6, 132.1, 128.1, 127.6, 127.5, 127.0, 126.5, 126.0, 125.4, 60.4, 35.9, 31.1, 14.2.

minor signals from **3j-*b***:[24]

^{1}H NMR (400 MHz, CDCl$_3$) δ 7.90-7.30 (m, 7H), 4.24-4.08 (m, 2H), 3.90 (q, *J* = 7.1 Hz, 1H), 1.62 (d, *J* = 7.2 Hz, 3H), 1.35-1.15 (m, 3H). ^{13}C NMR (100 MHz, CDCl$_3$) δ 174.6, 138.2, 133.5, 132.6, 128.3, 127.8, 127.7, 127.1, 126.2, 126.1, 125.8, 60.9, 45.7, 18.7, 14.2.

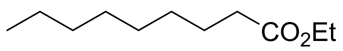

3k

Ethyl nonanoate (3k) (Table 5, entries 1-2): From 1-octene (**1k**) or *cis, trans*-2-octene (**1l**) (112.2 mg, 1.0 mmol), the title compound was prepared by following the general procedure I (100 °C, 24 h, Pd(acac)$_2$, EtOH) as a colorless oil in 98% yield for **1k** (182.6 mg) or in 95% yield for **1l** (177.0 mg). The spectroscopic data correspond to those previously reported in the literature.[30]

^{1}H NMR (400 MHz, $CDCl_3$) δ 4.12 (q, J = 7.1 Hz, 2H), 2.28 (t, J = 7.5 Hz, 2H), 1.68-1.55 (m, 2H), 1.26 (m, 13H), 0.88 (t, J = 6.2 Hz, 3H). ^{13}C NMR (100 MHz, $CDCl_3$) δ 173.9, 60.1, 34.4, 31.8, 29.2, 29.2, 29.1, 25.0, 22.6, 14.3, 14.1.

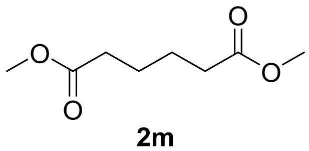

2m

Dimethyl adipate (2m) (Table 5, entry 3): From Methyl 2-pentenoate (**1m**) (114.1mg, 1.0 mmol), the title compound was prepared by following the general procedure I (100 °C, 24 h, $Pd(acac)_2$, MeOH) as a colorless oil in 75% yield (128.9 mg). The spectroscopic data correspond to those previously reported in the literature.[32]

^{1}H NMR (400 MHz, $CDCl_3$) δ 3.67 (s, 6H), 2.46-2.25 (m, 4H), 1.73-1.62 (m, 4H). ^{13}C NMR (100 MHz, $CDCl_3$) δ 173.7, 51.5, 33.6, 24.3.

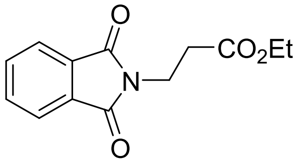

3n

Ethyl 3-phenylbutanoate (3n) (Table 5, entry 4): From 1-N-vinylphthalimide (**1n**) (86.6 mg, 0.5 mmol), the title compound was prepared by following the general procedure I (100 °C, 24 h, $Pd(acac)_2$, EtOH) as a white solid in 96% yield (118.7 mg). The spectroscopic data correspond to those previously reported in the literature.[30]

^{1}H NMR (400 MHz, $CDCl_3$) δ 7.90-7.81 (m, 2H), 7.78-7.67 (m, 2H), 4.13 (q, J = 7.1 Hz, 2H), 4.00 (t, J = 7.2 Hz, 2H), 2.73 (t, J = 7.2 Hz, 2H), 1.23 (t, J = 7.1 Hz, 3H). ^{13}C NMR (100 MHz, $CDCl_3$) δ 170.8, 168.0, 134.0, 132.1, 123.3, 60.8, 33.8, 33.0, 14.1.

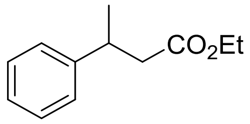

3o

Ethyl 3-phenylbutanoate (3o) (Table 5, entry 5): From 2-phenyl-1-propene (**1o**) (118.2 mg, 1.0 mmol), the title compound was prepared by following the general

procedure I (100 °C, 16 h, $Pd(OAc)_2$, EtOH) as a colorless oil in 35% yield (67.3 mg). The spectroscopic data correspond to those previously reported in the literature.[7,33]

^1H NMR (700 MHz, $CDCl_3$) δ 7.29 (t, J = 7.6 Hz, 2H), 7.24-7.18 (m, 3H), 4.07 (d, J = 7.1 Hz, 2H), 3.28 (d, J = 7.5 Hz, 1H), 2.57 (ddd, J = 23.2, 15.0, 7.6 Hz, 2H), 1.30 (d, J = 7.0 Hz, 3H), 1.18 (t, J = 7.1 Hz, 3H). ^{13}C NMR (175 MHz, $CDCl_3$) δ 172.4, 145.8, 128.5, 126.8, 126.4, 60.3, 43.0, 36.5, 21.8, 14.2.

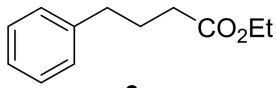

3p

Ethyl 4-phenylbutanoate (3p) (Table 5, entries 6-7): From allylbenzene (**1p**) or *trans*-β-Methylstyrene (**1q**) (118.2 mg, 1.0 mmol), the title compound was prepared by following the general procedure I (100 °C, 24 h, $Pd(acac)_2$, EtOH) as a colorless oil in 62% yield for **1p** (119.2 mg) or in 42% yield for **1q** (80.7 mg). The spectroscopic data correspond to those previously reported in the literature.[34]

^1H NMR (700 MHz, $CDCl_3$) δ 7.32-7.24 (m, 2H), 7.22-7.14 (m, 3H), 4.12 (q, J = 7.1 Hz, 2H), 2.65 (t, J = 7.6 Hz, 2H), 2.32 (t, J = 7.5 Hz, 2H), 2.03-1.90 (m, 2H), 1.25 (t, J = 7.1 Hz, 3H). ^{13}C NMR (100 MHz, $CDCl_3$) δ 173.5, 141.5, 128.5, 128.4, 126.0, 60.3, 35.2, 33.70, 26.6, 14.3.

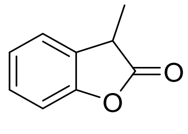

8a

3-Methylbenzofuran-2(3*H*)-one (8a) (Table 6, entry 1): From 2-vinylphenol (**7a**) (60.0 mg, 0.5 mmol), the title compound was prepared by following the general procedure II (60 °C, 48 h, $Pd(acac)_2$) as a yellowish white solid in 89% yield (65.9 mg). The spectroscopic data correspond to those previously reported in the literature.[2]

^1H NMR (400 MHz, $CDCl_3$) δ 7.35-7.22 (m, 2H), 7.19-7.06 (m, 2H), 3.73 (q, J = 7.5 Hz, 1H), 1.57 (d, J = 7.6 Hz, 3H). ^{13}C NMR (100 MHz, $CDCl_3$) δ 178.0, 153.5, 128.8, 128.8, 124.2, 123.9, 110.7, 38.4, 15.9.

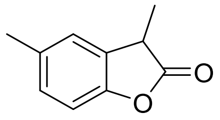

8b

3,5-Dimethylbenzofuran-2(3*H*)-one (8b) (Table 6, entry 2): From 4-methyl-2-vinylphenol (**7b**) (67.1 mg, 0.5 mmol), the title compound was prepared by following the general procedure II (60 °C, 48 h, Pd(acac)$_2$) as an oily white solid in 85% yield (68.9 mg). The spectroscopic data correspond to those previously reported in the literature.[35]

^{1}H NMR (400 MHz, CDCl$_3$) δ 7.15-7.02 (m, 2H), 7.00-6.90 (m, 1H), 3.68 (q, J = 7.4 Hz, 1H), 2.35 (s, 3H), 1.55 (d, J = 7.6 Hz, 3H). ^{13}C NMR (100 MHz, CDCl$_3$) δ 178.3, 151.4, 133.8, 129.1, 128.7, 124.4, 110.3, 38.5, 21.1, 15.9.

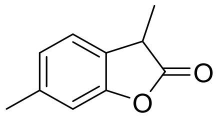

8c

3,6-Dimethylbenzofuran-2(3*H*)-one (8c) (Table 6, entry 3): From 5-methyl-2-vinylphenol (**7c**) (67.1 mg, 0.5 mmol), the title compound was prepared by following the general procedure II (60 °C, 48 h, Pd(acac)$_2$) as an oily white solid in 80% yield (64.9 mg). The spectroscopic data correspond to those previously reported in the literature.[2]

^{1}H NMR (700 MHz, CDCl$_3$) δ 7.12 (d, *J* = 7.6 Hz, 1H), 6.96 (d, *J* = 7.6 Hz, 1H), 6.93 (s, 1H), 3.69 (q, *J* = 7.6 Hz, 1H), 2.38 (s, 3H), 1.55 (d, *J* = 7.6 Hz, 3H). ^{13}C NMR (175 MHz, CDCl$_3$) δ 178.4, 153.6, 139.2, 125.7, 124.8, 123.5, 111.4, 38.3, 21.7, 16.0.

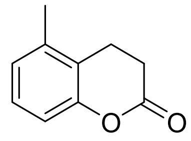

8d

5-Methylchroman-2-one (8d) (Table 6, entry 4): From 3-methyl-2-vinylphenol (**7d**) (67.1 mg, 0.5 mmol), the title compound was prepared by following the general procedure II (60 °C, 48 h, Pd(acac)$_2$) as an oily white solid in 68% yield (55.1 mg). The spectroscopic data correspond to those previously reported in the literature.[36]

^{1}H NMR (700 MHz, CDCl$_3$) δ 7.14 (t, *J* = 7.9 Hz, 1H), 6.97 (d, *J* = 7.6 Hz, 1H), 6.89 (d, *J* = 8.1 Hz, 1H), 2.93 (t, *J* = 7.4 Hz, 2H), 2.77 (t, *J* = 7.4 Hz, 2H), 2.31 (s, 3H). ^{13}C NMR (175 MHz, CDCl$_3$) δ 168.6, 152.1, 136.3, 127.7, 125.9, 121.2, 114.7, 28.8, 20.7, 19.2. HRMS (ESI+, m/z): calculated for $C_{10}H_{11}O_2$ [M+H] $^+$ 163.0759; found 163.0754.

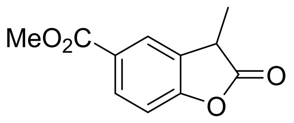

8e

Methyl 3-methyl-2-oxo-2,3-dihydrobenzofuran-5-carboxylate (8e) (Table 6, entry 5): From methyl 4-hydroxy-3-vinylbenzoate (**7e**) (89.0 mg, 0.5 mmol), the title compound was prepared by following the general procedure II (100 °C, 24 h, Pd(acac)$_2$) as an oily white solid in 80% yield (82.5 mg). The spectroscopic data correspond to those previously reported in the literature.[2]

^{1}H NMR (400 MHz, CDCl$_3$) δ 8.05 (d, *J* = 8.4 Hz, 1H), 7.97 (s, 1H), 7.16 (d, *J* = 8.4 Hz, 1H), 3.93 (s, 3H), 3.78 (q, *J* = 7.6 Hz, 1H), 1.61 (d, *J* = 7.6 Hz, 3H). ^{13}C NMR (100 MHz, CDCl$_3$) δ 177.1, 166.3, 156.9, 131.3, 129.0, 126.5, 125.6, 110.6, 52.3, 38.1, 15.8.

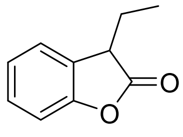

8f

3-Ethylbenzofuran-2(3*H*)-one (8f) (Table 6, entries 6-7): From 2-allylphenol (**7f**) (134.2mg, 1.0 mmol) or *β*-methyl-vinylphenol (**7g**) (67.1mg, 0.5 mmol), the title

compound was prepared by following general procedure II (100 °C, 16 h or 24 h, $Pd(acac)_2$) as a colorless oil in 78% yield (126.4 mg) for **7f** or in 37% yield (30.0 mg) for **7g**. The spectroscopic data correspond to those previously reported in the literature.[2]

^{1}H NMR (400 MHz, $CDCl_3$) δ 7.33-7.26 (m, 2H), 7.20-7.13 (m, 1H), 7.10 (d, *J* = 8.0 Hz, 1H), 3.71 (t, *J* = 5.9 Hz, 1H), 2.13-1.99 (m, 2H), 0.97 (t, *J* = 7.4 Hz, 3H). ^{13}C NMR (100 MHz, $CDCl_3$) δ 177.2, 153.9, 128.8, 127.2, 124.2, 124.1 110.7, 44.6, 24.3, 10.2.

7. References

1. N. Baret, J.-P. Dulcere, J. Rodriguez, J.-M. Pons and R. Faure, *Eur. J. Org. Chem.*, 2000, **2000**, 1507-1516.
2. V. Hirschbeck and I. Fleischer, *Chem. Eur. J.*, 2018, **24**, 2854-2857.
3. A. Seoane, N. Casanova, N. Quiñones, J. L. Mascareñas and M. Gulías, *J. Am. Chem. Soc.*, 2014, **136**, 834-837.
4. I. Fleischer and J. Pospech, *RSC Adv.*, 2015, **5**, 493-496.
5. R. P. Adams, P. S. Beauchamp, V. Dev and S. M. Dutz, *J. Essent. Oil Res.*, 2007, **19**, 146-152.
6. H. Konishi, T. Ueda, T. Muto and K. Manabe, *Org. Lett.*, 2012, **14**, 4722-4725.
7. S. S. Sohn and J. W. Bode, *Org. Lett.*, 2005, **7**, 3873-3876.
8. H. Nambu, K. Hata, M. Matsugi and Y. Kita, *Chem. Eur. J.*, 2005, **11**, 719-727.
9. C. Peng, W. Zhang, G. Yan and J. Wang, *Org. Lett.*, 2009, **11**, 1667-1670.
10. P. H. Gehrtz, V. Hirschbeck and I. Fleischer, *Chem. Commun.*, 2015, **51**, 12574-12577.
11. M. D. Hossain and T. Kitamura, *Synthesis*, 2006, **2006**, 1253-1256.
12. J. M. Concellón and M. Huerta, *J. Org. Chem.*, 2005, **70**, 4714-4719.
13. M. Amatore, C. Gosmini and J. Périchon, *J. Org. Chem.*, 2006, **71**, 6130-6134.
14. C. Liu, C. He, W. Shi, M. Chen and A. Lei, *Org. Lett.*, 2007, **9**, 5601-5604.
15. R. Mueller, J. Yang, C. Duan, E. Pop, O. J. Geoffroy, L. H. Zhang, T.-B. Huang, S. Denisenko, B. H. McCosar, D. C. Oniciu, C. L. Bisgaier, M. E. Pape, C. D. Freiman, B. Goetz, C. T. Cramer, K. L. Hopson and J.-L. H. Dasseux, *J. Med. Chem.*, 2004, **47**, 6082-6099.
16. Y. Kita, Y. Nishii, A. Onoue and K. Mashima, *Adv. Synth. Catal.*, 2013, **355**, 3391-3395.
17. A. Grabulosa, J. J. R. Frew, J. A. Fuentes, A. M. Z. Slawin and M. L. Clarke, *J. Mol. Catal. A: Chem.*, 2010, **330**, 18-25.
18. C. Salomé and H. Kohn, *Tetrahedron*, 2009, **65**, 456-460.
19. S. Chandrasekhar, G. Pavan Kumar Reddy, C. Nagesh and C. Raji Reddy, *Tetrahedron Lett.*, 2007, **48**, 1269-1271.
20. G. S. Coumbarides, M. Dingjan, J. Eames, A. Flinn and J. Northen, *J. Labelled Compd. Radiopharm.*, 2006, **49**, 903-914.
21. D. Leow, G. Li, T.-S. Mei and J.-Q. Yu, *Nature*, 2012, **486**, 518-522.
22. B. A. Sandoval, A. J. Meichan and T. K. Hyster, *J. Am. Chem. Soc.*, 2017, **139**, 11313-11316.
23. E. W. H. Ng, K.-H. Low and P. Chiu, *J. Am. Chem. Soc.*, 2018, **140**, 3537-3541.
24. Y.-S. Feng, W. Wu, Z.-Q. Xu, Y. Li, M. Li and H.-J. Xu, *Tetrahedron*, 2012, **68**, 2113-2120.
25. X.-S. Zhang, Q.-L. Zhu, Y.-F. Zhang, Y.-B. Li and Z.-J. Shi, *Chem. Eur. J.*, 2013, **19**, 11898-11903.
26. N. A. Strotman, S. Sommer and G. C. Fu, *Angew. Chem. Int. Ed.,* 2007, **46**, 3556 –3558.
27. B. M. Nestl, S. M. Glueck, M. Hall, W. Kroutil, R. Stuermer, B. Hauer and K. Faber, *Eur. J. Org. Chem.*, 2006, **2006**, 4573-4577.
28. S. Condon, D. Dupré, G. Falgayrac and J.-Y. Nédélec, *Eur. J. Org. Chem.*, 2002, **2002**, 105-111.
29. M. Zysk, A. Zadlo, A. Brodzka, C. Wisniewska and R. Ostaszewski, *J. Mol. Catal. B: Enzym.*, 2014, **102**, 225-229.
30. Z. Deng, S. Han, M. Ke, Y. Ning and F.-E. Chen, *Chem. Commun.*, 2022, **58**, 3921-3924.
31. C. Hardouin, M. J. Kelso, F. A. Romero, T. J. Rayl, D. Leung, I. Hwang, B. F. Cravatt and D. L. Boger, *J. Med. Chem.*, 2007, **50**, 3359-3368.

32. I. Fleischer, R. Jennerjahn, D. Cozzula, R. Jackstell, R. Franke and M. Beller, *ChemSusChem*, 2013, **6**, 417-420.
33. C. Metallinos, J. Zaifman, L. Van Belle, L. Dodge and M. Pilkington, *Organometallics*, 2009, **28**, 4534-4543.
34. D. A. Everson, R. Shrestha and D. J. Weix, *J. Am. Chem. Soc.*, 2010, **132**, 920-921.
35. M. Wang, X. Zhang, Z. Ling, Z. Zhang and W. Zhang, *Chem. Commun.*, 2017, **53**, 1381-1384.
36. Y. Gu and K. Xue, *Tetrahedron Lett.*, 2010, **51**, 192-196.

8. NMR Spectra

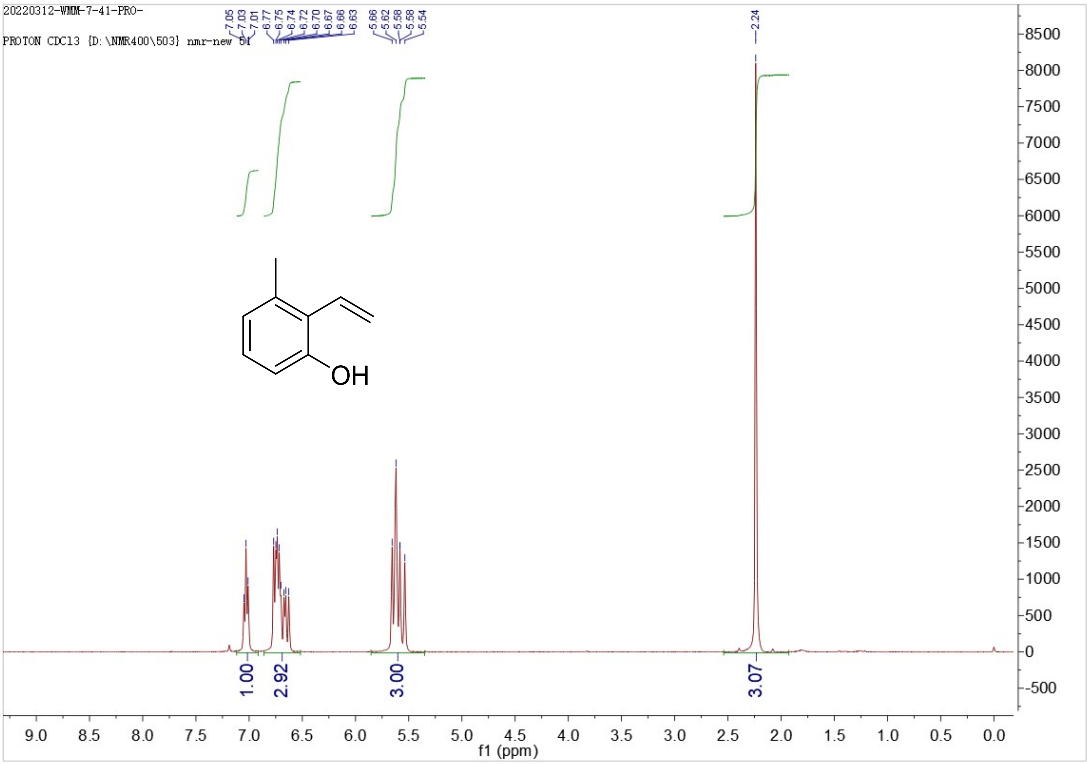

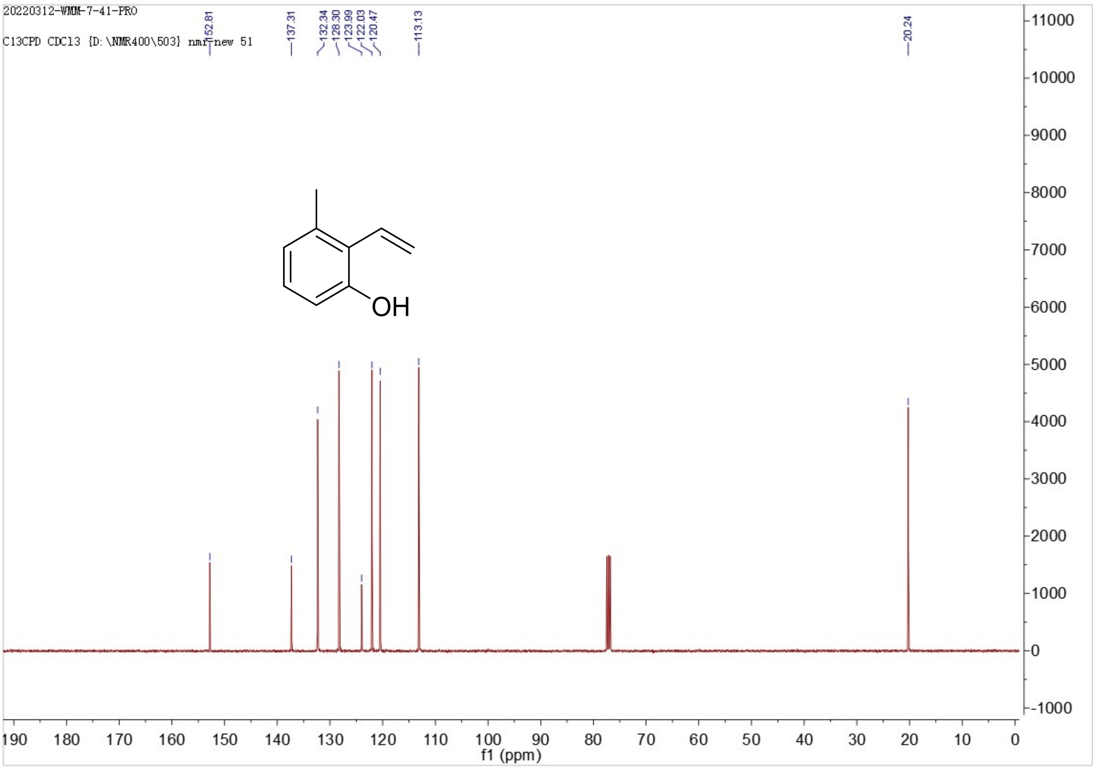

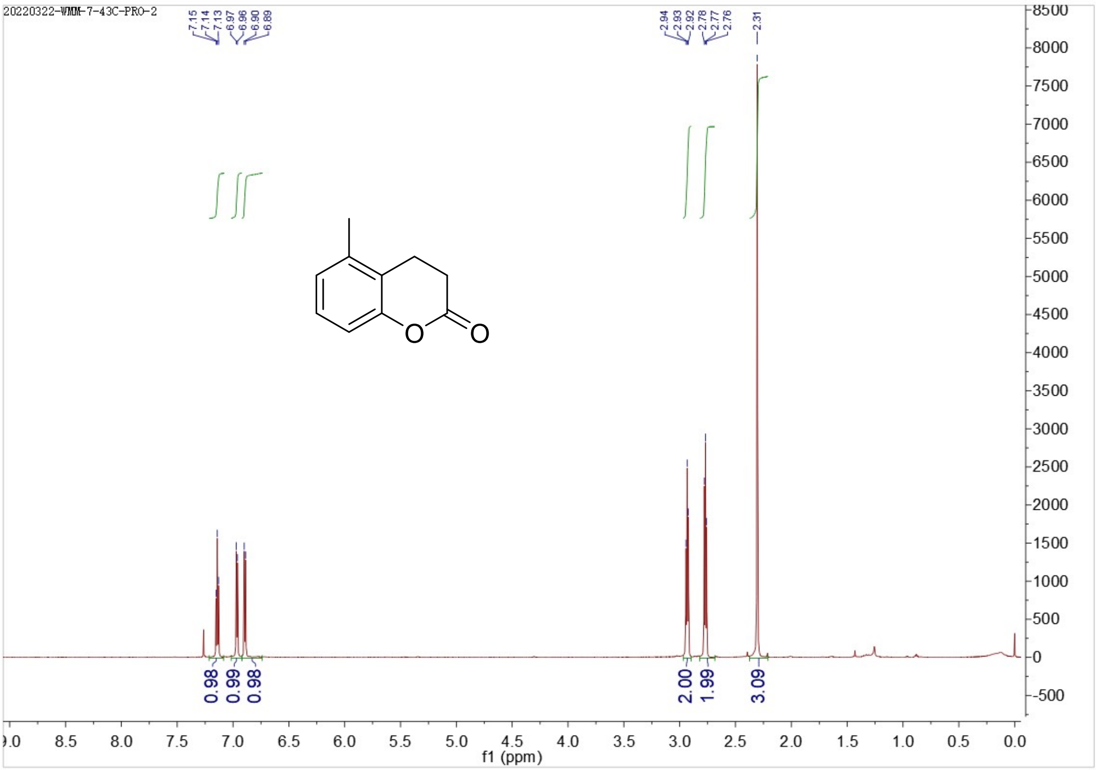
20220322-WMM-7-43C-PRO-2
7.15
7.14
7.13
6.97
6.96
6.90
6.89
2.94
2.93
2.92
2.78
2.77
2.76
2.31
0.98
0.99
0.98
2.00
1.99
3.09
O
O
f1 (ppm)

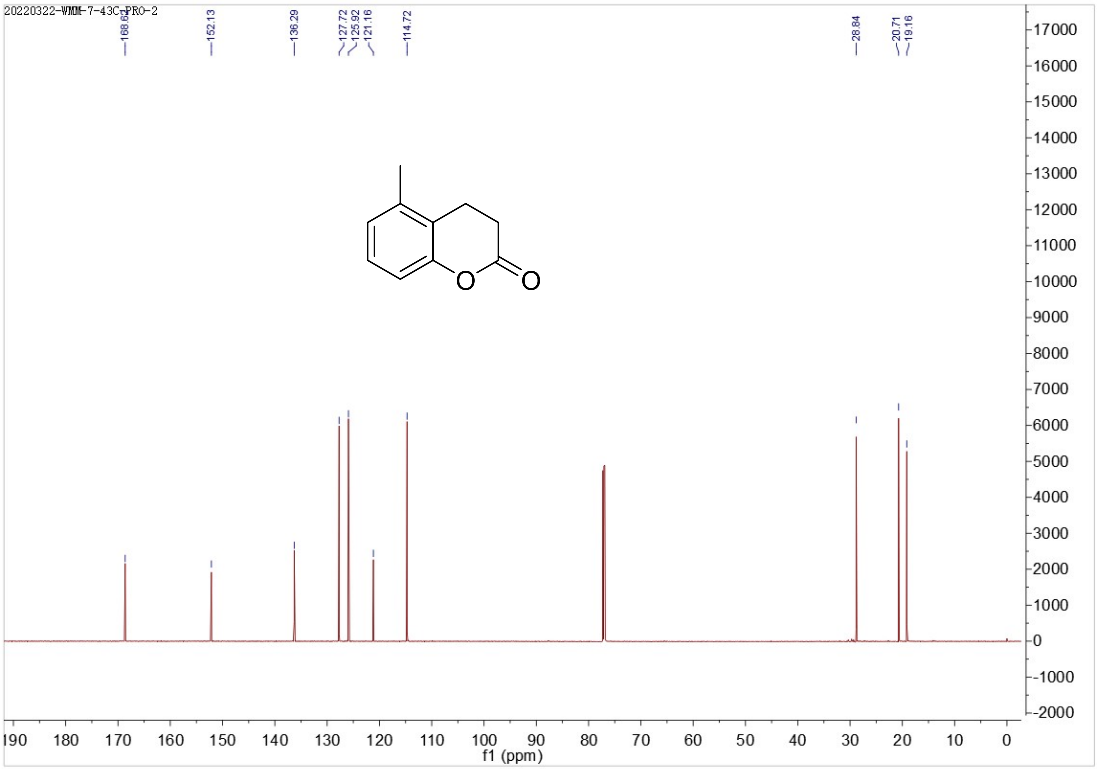
20220322-WMM-7-43C-PRO-2
152.13
136.29
127.72
125.92
121.16
114.72
28.84
20.71
19.16
O
O
f1 (ppm)
